# The role of the *Aspergillus nidulans* high mobility group B protein HmbA, the orthologue of *Saccharomyces cerevisiae* Nhp6p

**DOI:** 10.1038/s41598-022-22202-3

**Published:** 2022-10-15

**Authors:** Judit Ámon, Gabriella Varga, Ilona Pfeiffer, Zoltán Farkas, Zoltán Karácsony, Zsófia Hegedűs, Csaba Vágvölgyi, Zsuzsanna Hamari

**Affiliations:** 1 Department of Microbiology, Faculty of Science and Informatics, University of Szeged, Szeged, Hungary; 2 Synthetic and Systems Biology Unit, Institute of Biochemistry, Biological Research Centre, Eötvös Loránd Research Network, Szeged, Hungary; 3 Present Address: Food and Wine Research Institute, Eszterházy Károly University, Eger, Hungary

**Keywords:** Microbiology, Fungi, Fungal biology, Fungal genetics, Fungal physiology

## Abstract

The mammalian HMGB1 is a high-mobility-group B protein, which is both an architectural and functional element of chromatin. Nhp6p, the extensively studied fungal homologue of HMGB1 in *Saccharomyces cerevisiae* has pleiotropic physiological functions. Despite the existence of Nhp6p orthologues in filamentous ascomycetes, little is known about their physiological roles besides their contribution to sexual development. Here we study the function of HmbA, the *Aspergillus nidulans* orthologue of Nhp6p. We show that HmbA influences the utilization of various carbon- and nitrogen sources, stress tolerance, secondary metabolism, hyphae elongation and maintenance of polarized growth. Additionally, by conducting heterologous expression studies, we demonstrate that HmbA and Nhp6p are partially interchangeable. HmbA restores *SNR6* transcription and fitness of *nhp6AΔBΔ* mutant and reverses its heat sensitivity. Nhp6Ap complements several phenotypes of *hmbAΔ*, including ascospore formation, utilization of various carbon- and nitrogen-sources, radial growth rate, hypha elongation by polarized growth. However, Nhp6Ap does not complement sterigmatocystin production in a *hmbAΔ* strain. Finally, we also show that HmbA is necessary for the normal expression of the endochitinase *chiA*, a cell wall re-modeller that is pivotal for the normal mode of maintenance of polar growth.

## Introduction

Chromatin structuring high-mobility-group B (HMG-B) proteins are not only architectural but also functional elements of chromatin (reviewed in^1^). Architectural HMG-B proteins typically harbour two or more copies of HMG-box domains interacting with DNA in a non-sequence-specific manner together with various protein components of chromatin. They are not only structural components of chromatin but they facilitate the establishment of stable protein-DNA interactions between chromatin-associated activator or repressor proteins and their cognate DNA motif, contributing to the normal functioning of chromatin (reviewed in^2^).

The non-conventional homologues of mammalian HMGB1 (earlier HMG1/2), the Nhp6Ap and Nhp6Bp paralogues from *Saccharomyces cerevisiae*^3,4^ were the first architectural HMG-B proteins studied in fungi. Unlike most HMGB proteins, these paralogue proteins include only one copy of the HMG-box domain and a short, basic N-terminal tail. The basic N-terminal region establishes electrostatic interactions with the DNA phosphodiester backbone of the major groove while the L-shaped three helices of the HMG-box domain bind into the minor groove via both electrostatic and hydrophobic interactions^5,6^. These protein-DNA interactions result in a sharp bend of the DNA backbone or stabilize an already distorted or non-B-type DNA^6,7^. These interactions with DNA, as well as the establishment of protein–protein interactions with other chromatin-associated proteins, allow the Nhp6 proteins to promote nucleosome assembly, and the formation of DNA–protein complexes including various transcription factors, activators, remodelling complexes or repressors^7–11^. Nhp6 proteins participate in the activation of transcriptions by RNA polymerase II and the activation of the U6 spliceosomal RNA coding gene (*SNR6*) by RNA polymerase III^7,12–14^. Transcriptome analyses of both *NHP6*^+^ and *nhp6AΔBΔ* strains revealed that the absence of Nhp6 proteins has a pleiotropic effect, as the expression of several hundred genes is altered in the *nhp6AΔBΔ* strain^14,15^. The physiological role of the Nhp6 proteins was previously investigated by using *NHP6A/NHP6B* double deletion mutants (*nhp6AΔBΔ*)^16^. The phenotype of *nhp6AΔBΔ* was found to be similar to that of an *Slt2Δ* mutant (deleted for the stress-activated MAP kinase coding *SLT2*, a component of the Slt2/Mpk1 MAPK signal transduction route that functions in cell morphogenesis and growth control) and both *NHP6A* and *NHP6B* act individually as multicopy suppressors of *Slt2Δ*^16^. Furthermore, an *nhp6AΔBΔ* strain has reduced growth and various morphological abnormalities, is sensitive to both nitrogen-starvation and 38 °C, a phenotype that can be suppressed by supplementing the medium with 1 M sorbitol^16^.

Although filamentous Ascomycete fungi also have orthologues of the *Saccharomyces cerevisiae* Nhp6p proteins, their physiological functions, besides from their role in sexual development has not been addressed. Both PaHMG6 and HmbA, which are orthologues of *S. cerevisiae* Nhp6p proteins from *Podospora anserina* and *Aspergillus nidulans*, respectively, are required for the proper expression of the mating-type genes^17,18^. In *A. nidulans*, the *hmbA* gene deletion mutants can produce cleistothecia, which do not contain ascospores ^18^. Despite these observations, the overall physiological function of HmbA remained unexplored. Here we thoroughly examined the physiological functions of HmbA by analysing the utilization of various carbon- and nitrogen sources, secondary metabolism, stress sensitivity and micromorphology-related phenotypes in an *hmbA* deletion mutant. Furthermore, we investigated the functional interchangeability of HmbA and Nhp6Ap by heterologous expression of *hmbA* and *NHP6A* genes in *S. cerevisiae nhp6AΔBΔ* and *A. nidulans hmbAΔ* mutants, respectively.

## Results and discussion

### HmbA is structurally similar to Nhp6Ap

**Figure 1:**
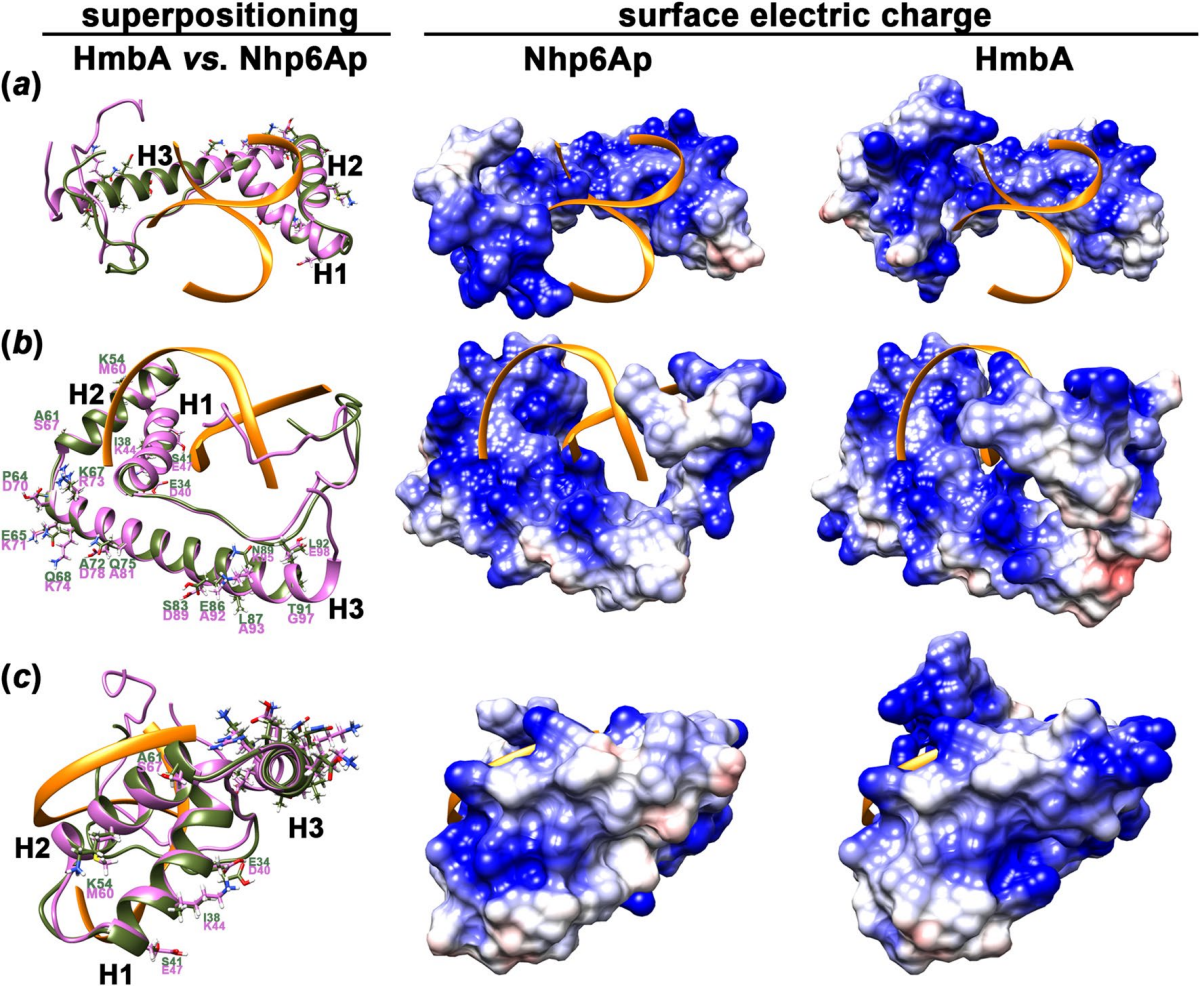
Comparison of the structure and electrostatic surface topography between HmbA and Nhp6Ap. Both models were generated in this work (see “Methods” Section). The yellow ribbon depicts the DNA ligand. The DNA was positioned by superimposing of the NMR-based model structure of Nhp6Ap-DNA complex (PDB ID: 1cg7) with the HmbA and Nhp6Ap models generated in this work followed by hiding the NMR-based Nhp6Ap model and keeping the DNA ligand visible. The left side of each panel shows the superimposition of HmbA (pink ribbon) with Nhp6Ap (olive ribbon) from different angles, whereas the middle and right sides of each panel correspond to Nhp6Ap and HmbA, respectively and show the electrostatic potential of the exposed molecule parts. AA residues that differ between the helices of HmbA and Nhp6Ap are indicated by showing their side chains in stick view. (**a**) The DNA binding interface is shown. None of the altering AA residues map to the DNA binding interface. (**b**) H2 and H3 helices are shown as well as the N-termini and the negatively charged C-terminus that is characteristic only to HmbA. (**c**) H1 and H2 helices are shown. H3 helix is shown from a top view, which illuminates that the altering AA residues are positioned to the surface of the molecule. H1, H2 and H3 denote helices from N to C termini. Electrostatic potential from -10 (red colour) to + 10 (blue colour) of the modelled proteins is shown. White colour indicates uncharged area.

We first investigated the structural similarities of HmbA and Nhp6Ap. An NMR-based model structure of the Nhp6Ap-DNA complex is available^6^, however, for comparative purposes we generated both ligand-free Nhp6Ap and HmbA models (Fig. 1, Supplementary Table 1). The two proteins show a 67.74% amino acid (AA) identity and the three dimensional structures of the proteins overlap, especially at the three alpha-helices (H1–H3) (Supplementary Table S1 and Fig. 1). The HmbA and Nhp6Ap H1 and H2 helices are highly similar (13 and 12 AAs are identical out of the 16 and 14 AA long helices, respectively), while the H3 helices are less similar (only 10 AAs out of the total 29 AAs of Nhp6Ap H3 helix are identical in HmbA) (Fig. 1). Notably, none of the differing AA residues of the helices map to the DNA binding interface, but to the outer surface of the molecule (Fig. 1a). These AA differences between HmbA and Nhp6Ap result in different surface electrostatic topography, which might be a determinant property for the establishment of interactions with other chromatin proteins (Fig. 1). Absolute conservation of the DNA-binding interface in HmbA and Nhp6Ap includes the (i) R19-K22 (RKKK) region, responsible for binding to the major groove (R13-K16 in Nhp6Ap); (ii) P27 (P21 in Nhp6Ap) that directs the N-terminal tail toward the major groove^5^; (iii) S32 and Y34 (S26 and Y28 in Nhp6Ap) that establish H-bonds with the bases of the minor groove and are supposed to be responsible for selectivity for a T-G or G-T dinucleotide sequence^6^; and (iv) M35 and F54 (M29 and F48 in Nhp6Ap) that intercalate into the major groove^5,6^. The above findings strongly suggest the DNA binding properties of the *S. cerevisiae* and *A. nidulans* HMG-B proteins are similar, while their ability to interact with other protein components of the chromatin may well be different.

However, if the interaction was dependent on the surface AAs of the most conserved H1 and H2 helices (Fig. 1b,c), we could not rule out common interacting protein partners of HmbA and Nhp6Ap. The *S. cerevisiae* and *A. nidulans* proteins are strikingly different at the C-terminal end. The HmbA protein ends in a negatively charged unstructured tail that might have a specific functional role, a sequence that is absent in Nhp6Ap (its carboxy terminal ends with the H3 helix) (Fig. 1b).

### HmbA can complement Nhp6Ap functions

**Figure 2:**
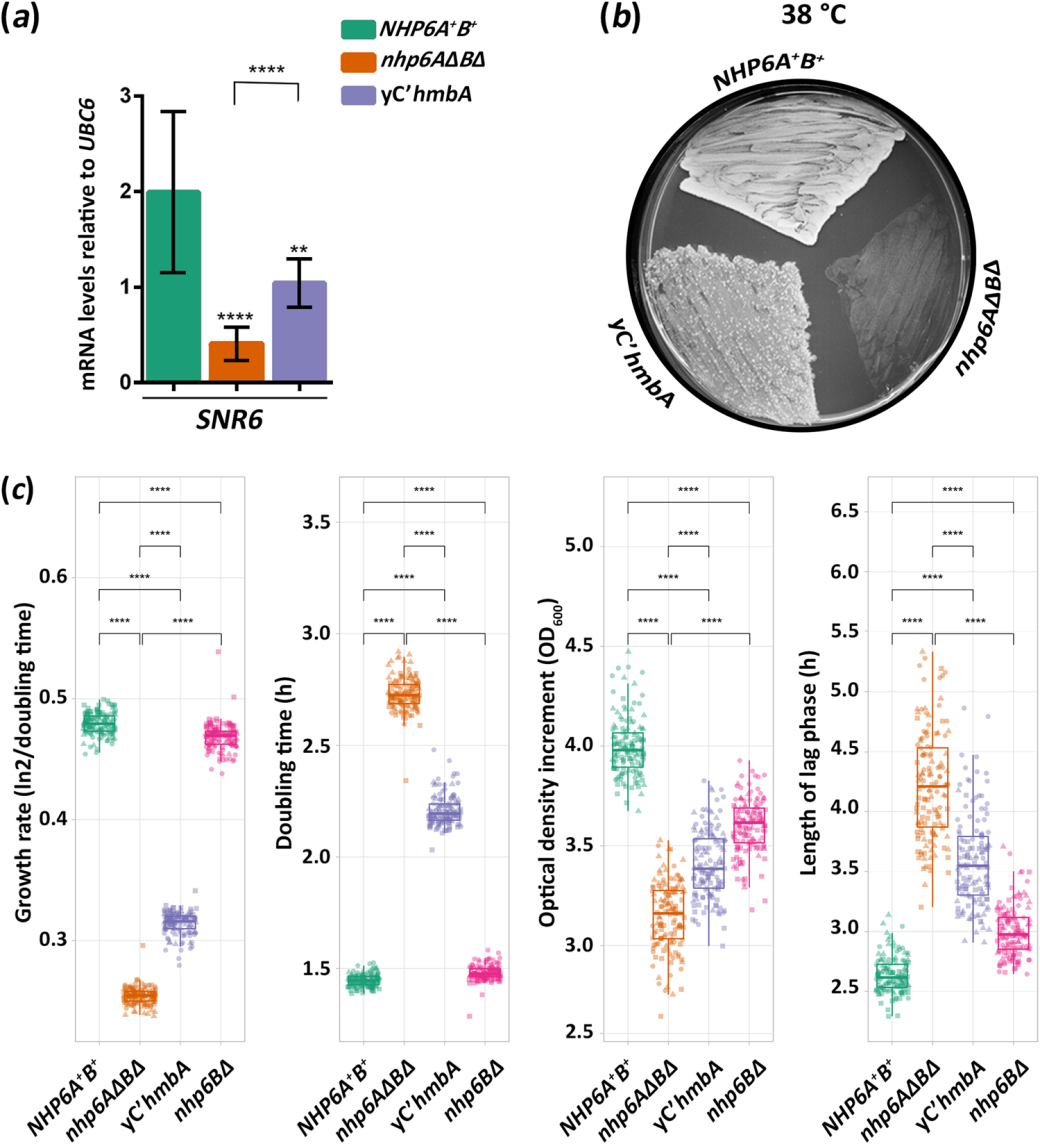
Heterologous expression of *A. nidulans hmbA* rescues the downregulation of *SNR6*, heat sensitivity and reduced fitness of the *nhp6AΔBΔ* strain. (**a**) mRNA levels of *SNR6* in *NHP6A*^+^*B*^+^control (Y199), *nhp6AΔBΔ* (HZS.891) and yC’*hmbA* (*nhp6AΔBΔ* complemented with *hmbA*, HZS.890) strains measured by RT-qPCR. Strains were grown on YPD up to OD_600_ = 0.25 at 30 °C followed by 1 h additional incubation at 38 °C. RT-qPCR data were processed according to the standard curve method ^44^ with *UBC6* as the reference mRNA. The mean and standard deviations of three independent experiments are shown. Significance between strains was assessed by using Student’s *t*-test. **/**** indicate *P* < 0.01/0.0001. Complete genotypes of the strains and used primers are listed in Supplementary Table S2 and S4, respectively. (**b**) Heterologous expression of *A. nidulans hmbA* reversed the heat sensitive phenotype of *nhp6AΔBΔ*. Strains (same as described in panel *a* were grown at 38 °C on YPD. (**c**) Results of fitness measurements including growth rate, doubling time, optical density increment, and length of the lag phase. Strains are the same as described in panel *a* except that *nhp6BΔ* strain was included in the fitness experiment as additional control. Complete genotypes are listed in Supplementary Table S2. Fitness measurements were done in three biological replicates with 45 technical replicates per each experiment. Triangles, squares and circles denote the three biological replicates. Significance between strains was assessed by using Mann–Whitney U-test. **** indicates *P* < 0.0001.

We investigated the functional similarities of HmbA and Nhp6Ap by complementation studies. Proper expression of *SNR6* gene in *S. cerevisiae* depends on the Nhp6p proteins^12,13^. Interaction of Nhp6Ap with the *SNR6* promoter facilitates the binding of TFIIIC to its sub-optimally spaced *SNR6* promoter elements^13^. While the absence of Nhp6p proteins is tolerated at 30 °C (TFIIIC can bind to the *SNR6* promoter elements, although with a reduced level), the binding of TFIIIC to the sub-optimally spaced *SNR6* promoter elements at 38 °C does not occur in *nhp6AΔBΔ* strains^13^. The *SNR6* gene expression is downregulated in an *nhp6AΔBΔ* strain at 30 °C, and it is completely absent at 38 °C^13^. The *nhp6AΔBΔ* mutant shows a reduced growth at 30 °C, whereas it is non-viable at 38 °C^12,13,16^. The heterologous expression of the *A. nidulans hmbA* from the native *NHP6A* promoter in the *nhp6AΔBΔ* strain (hereafter referred to as yC’*hmbA*) significantly increased *SNR6* expression and reversed the heat sensitive phenotype of *nhp6AΔBΔ* (Fig. 2a,b), similarly to the *nhp6AΔBΔ* strain complemented by *NHP6A*, with the difference that *SNR6* expression was completely restored to the wild type level by *NHP6A*^13,16^.

Other *nhp6AΔBΔ* phenotypes, such as prolonged doubling time, were also investigated in a yC’*hmbA* strain and an *NHP6A nhp6BΔ* strain, which should be equivalent to an *nhp6AΔBΔ* complemented with *NHP6A*. We found that the doubling time and other fitness parameters, such as growth rate, optical density increment and length of lag phase, were also suppressed by *hmbA* in an *nhp6AΔBΔ* strain (Fig. 2c). Although the complementation was not complete, the yC’*hmbA* strain showed significant improvement in all tested fitness components (*P* < 0.0001). The *NHP6A nhp6BΔ* strain showed nearly wild-type like growth rate and doubling time, however, the deviation from the wild-type levels was significant in all fitness components tested (*P* < 0.0001).

### Heterologous expression of *NHP6A* in the *hmbAΔ* strain restores the ascospore production in the fruiting bodies

**Figure 3:**
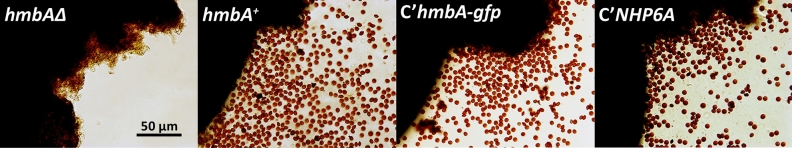
Ascospore content of *hmbAΔ*, *hmbA*^+^ control and various complemented *hmbAΔ* strains. Images show crushed cleistothecia releasing red ascospores. Cleistothecia were collected from self-fertilized 14 days old colonies grown on complete medium. Scale bar is shown. The strains used in the analysis were *hmbAΔ* (*hmbA* deletion strain, HZS.320), *hmbA*^+^ as control (HZS.120); C’*hmbA-gfp* (*hmbAΔ* complemented with *hmbA-gfp*, HZS.371) and C’*NHP6A* (*hmbAΔ* complemented with *NHP6A*, HZS.834). Complete genotypes are listed in Supplementary Table S2. Images were taken by an Olympus BX51 microscope.

To investigate the role of Nhp6Ap in ascospore production, we constructed an *hmbAΔ* strain complemented by *NHP6A* (hereafter referred to as C’*NHP6A*), in which *NHP6A*, the yeast orthologue of *hmbA,* is expressed from the *hmbA* promoter (P_*hmbA*_) (for details see “Methods” Section). Microscopy analysis of the content of crushed C’*NHP6A* cleistothecia showed that the yeast gene restores the ascospore formation in the *hmbAΔ* strain (Fig. 3). An *hmbAΔ* strain expressing a HmbA-Gfp fusion protein (C’*hmbA-gfp,* the gene fusion was expressed from the constitutive promoter of *gpdA*) also fully complemented the deletion phenotype (Fig. 3). A restoration of the wild-type phenotype was reported by us earlier in an *hmbA* complemented *hmbAΔ* strain (called as C’*hmbA*), in which *hmbA* was expressed under the control of its physiological promoter (P_*hmbA*_)^18^.

### *hmbA* is steadily expressed from germination to mycelial growth and the encoded protein localizes in the nucleus

**Figure 4:**
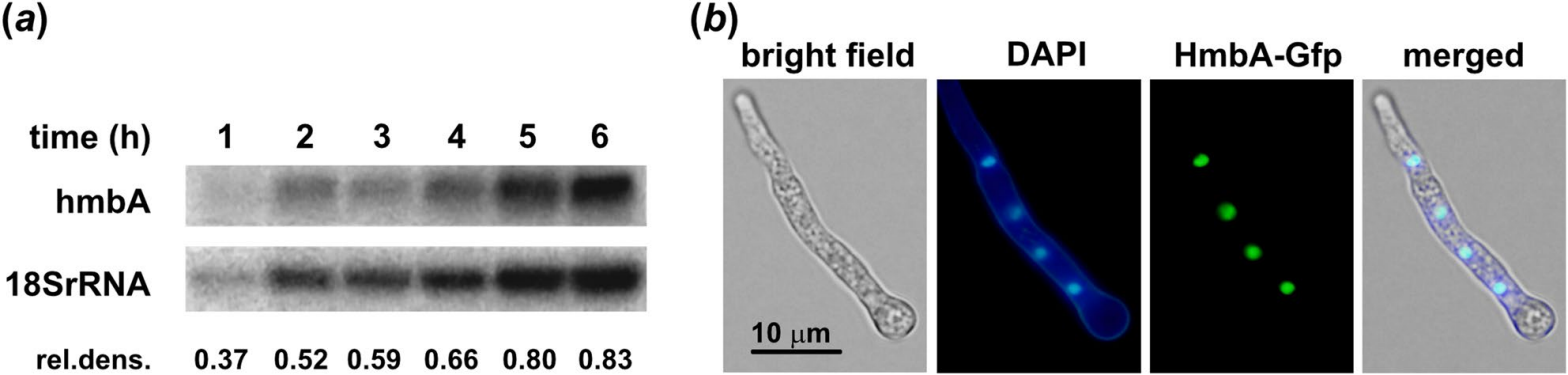
Expression profile of *hmbA* and intracellular localization of the gene product. (**a**) Northern blot analysis of the mRNA level of *hmbA* from germination to mycelial growth (1–6 h) in the *hmbA*^+^ strain HZS.117. Loading of RNA was estimated by hybridization with an 18S rRNA specific DNA probe. *hmbA* mRNA levels relative to that of the housekeeping *18S rRNA* gene were calculated by normalization of the optical density of each amplified *hmbA* band shown in Northern blot to that of the *18S rRNA* gene by using ImageJ. Used medium was MM with 5 mM urea as sole nitrogen-source. (**b**) Subcellular localization of HmbA-Gfp with DNA staining (DAPI) of young hypha in C’*hmbA-gfp* strain (HZS.371). Conidiospores were germinated for 8 h in MM at 37 °C. Cells were stained for DNA (DAPI) and examined by fluorescence microscopy (Zeiss 49 and 15 filter sets were used for DAPI and Gfp, respectively). Scale bar represents 10 μm. Complete genotypes are listed in Supplementary Table S2.

To investigate the *hmbA* expression, we measured the mRNA levels of *hmbA* in a *hmbA*^+^ strain by Northern blots (see “Methods” Section). We found that the mRNA levels of *hmbA* showed positive correlation with the level of 18S rRNA during development (from germination to the mycelial growth) (Fig. 4a). In addition, we sought to confirm the nuclear localization of HmbA. The HmbA-Gfp protein co-localized with the DAPI stained nuclei (Fig. 4b).

### HmbA influences both the utilization of various carbon-/nitrogen-sources and the response to environmental stresses

**Figure 5:**
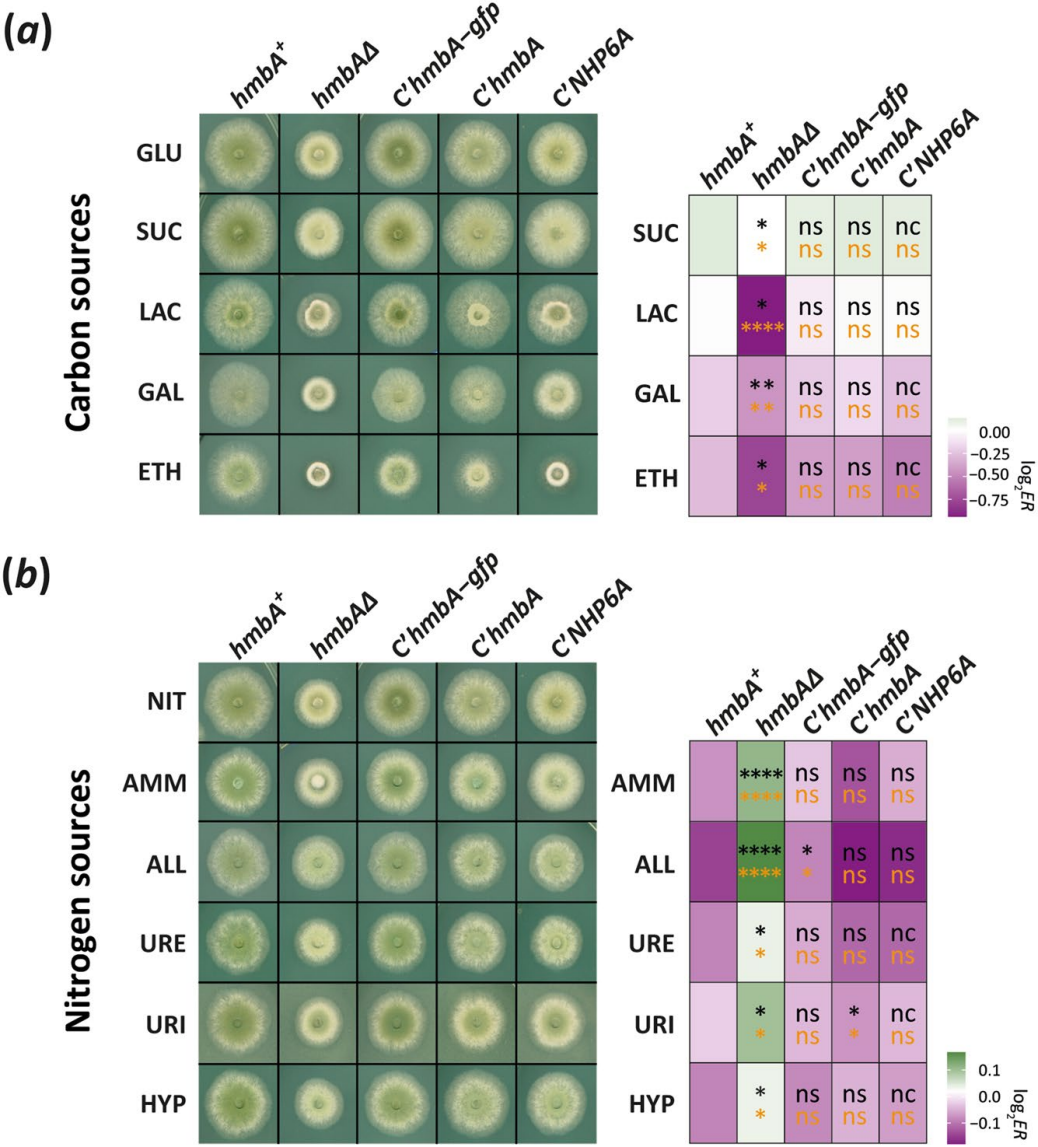

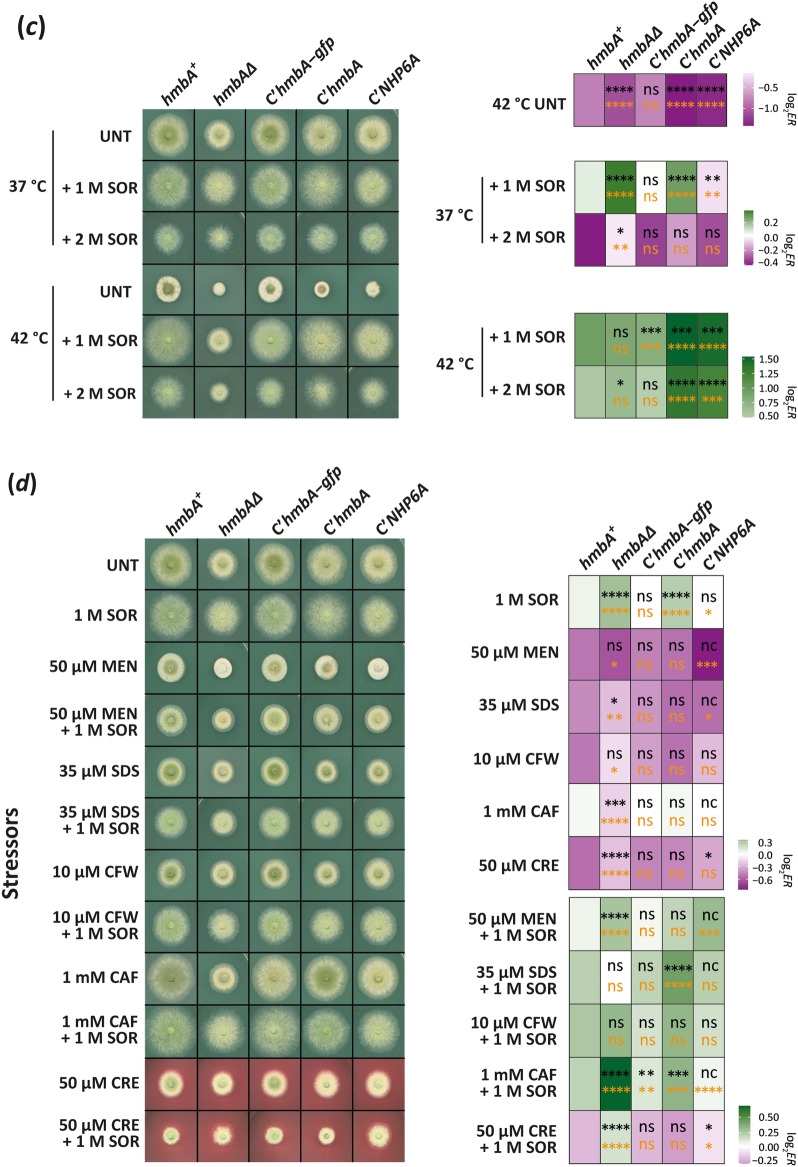
Growth tests and *ER* values of *hmbA*^+^, *hmbAΔ* and complemented control strains on various carbon- and nitrogen-sources and under various environmental stress conditions. On the right side of each panel, the heatmap shows the log_2_ *ER* values (green and magenta indicates increase and decrease, respectively). Asterisks in the cells show the significance of Student’s *t*-test (orange) and Mann–Whitney U-test (black) calculated by comparing the *ER* values of all tested strains to that of the *hmbA*^+^ control (*/**/***/**** indicates *P* < 0.05, 0.01, 0.001 and 0.0001, respectively; nc: non-calculated due to low sample size, ns: non-significant). Raw data and the calculated *ER* values can be found in Supplementary Dataset File. (**a**) Growth tests on various carbon-sources, including GLU (glucose), SUC (sucrose), LAC (lactose), GAL (galactose) and ETH (ethanol). The nitrogen-source was sodium nitrate in all conditions. (**b**) Growth tests on various nitrogen-sources, including NIT (sodium-nitrate), AMM (diammonium L-(+)-tartrate), ALL (allantoin), URE (urea), URI (uric acid) and HYP (hypoxanthine) as sole nitrogen-sources. The carbon-source was glucose in all conditions. (**c**) Growth tests under heat-stress (42 °C instead of the normal 37 °C) without (UNT, untreated) or in the presence of 1 M sorbitol (1 M SOR) as osmotic stabilizer or 2 M sorbitol (2 M SOR) as osmotic stressor. All medium used in the test was a glucose-sodium nitrate minimal medium (MM). (**d**) Growth tests on glucose-sodium nitrate MM (UNT, untreated) supplemented with various stress agents in the indicated concentrations without or in the presence of 1 M sorbitol (1 M SOR) osmotic stabilizer. MEN, menadione; SDS, sodium dodecyl sulphate; CFW, Calcofluor White; CAF, caffeine; CR, congo red. Used strains were *hmbA*^+^ as control (HZS.120); *hmbAΔ* (*hmbA* deletion strain, HZS.320); *C’hmbA* (*hmbAΔ* complemented with *hmbA*, HZS.621), C’*hmbA-gfp* (*hmbAΔ* complemented with *hmbA-gfp*, HZS.371); C’*NHP6A* (*hmbAΔ* complemented with *NHP6A*, HZS.834). Genotypes of the strains are listed in Supplementary Table S2.

Deletion of *hmbA* results in reduced growth on complete medium due to the decrease in the elongation rate of the hyphae^18^. To further investigate the growth characteristics and environmental responses of *hmbAΔ* and various complemented strains, we performed growth tests on a large variety of conditions (left panels of Fig. 5). The growth tests were performed using glucose-nitrate medium at 37 °C as a control condition. On this medium *hmbAΔ* showed a significantly reduced growth (*P* < 0.0001, Mann–Whitney U-test) compared to both the *hmbA*^+^ and all types of complemented strains (Supplementary Fig. S1*a*). In order to assess significant differences in the environmental response (*ER*) of the examined strains to the tested conditions, we controlled for the intrinsic differences in growth between the various strains by using strainwise normalization of the raw colony sizes observed in the tested condition to the average colony size in the control condition (*ER* = size_tested condition_/average size_control condition_, Supplementary Dataset File). Significantly higher and lower *ER* values between the deletion/complemented strains and the *hmbA*^+^ control strain indicate positive and negative growth change, respectively. The *ER* values in all selected conditions are presented in Supplementary Fig. S1, whereas the log_2_*ER* values compared to the control conditions are presented in the right panels of Fig. 5.

First, we investigated the response to changing the carbon source from glucose to 9 various carbon-sources (Supplementary Fig. S1*b*). The *ER* was significantly decreased (*P* < 0.05–0.001) in *hmbAΔ* compared to *hmbA*^+^ control on medium containing sucrose, lactose, galactose and ethanol as sole carbon-sources (heatmap of Fig. 5a, Supplementary Fig. S1*b*). We did not find any significant differences in the *ER* on the other tested carbons sources, including glycine, maltose, raffinose, sorbitol and xylose (Supplementary Fig. S1*b*).

Next, the *ER* to 6 different nitrogen-sources was investigated (heatmap of Fig. 5b, Supplementary Fig. S1*c*). The only non-significant difference between the *ER* of *hmbA*^+^ and *hmbAΔ* was in the presence of acetamide as sole nitrogen-source (Supplementary Fig. S1*c*). In all other cases, including diammonium L-(+)-tartrate, allantoin, urea, uric acid and hypoxanthine as sole nitrogen-sources, the *ER* of *hmbAΔ* was significantly higher (*P* < 0.05–0.0001) than that of the *hmbA*^+^ control.

This unexpected large number of nitrogen sources with increased *ER* values in *hmbAΔ* might be biased by the arbitrary selection of the control nitrogen source (nitrate), therefore we repeated the analysis by using the glucose-ammonium as control condition (Supplementary Fig. S1*d*). From this alternative point-of-view, significantly reduced ER value in *hmbAΔ* compared to *hmbA*^+^ was observed only on nitrate (*P* < 0.0001), whereas significantly increased *ER* value was observed only on allantoin (*P* < 0.0001). Using the ammonium normalized *ER* values, the *hmbAΔ* responded similarly to the change of the N-source to urea, uric acid, acetamide and hypoxanthine as the *hmbA*^+^ control did (Supplementary Fig. S1*d*).

The *ER* values of the complemented strains to all alternative carbon- and nitrogen-sources was not significantly different from the *hmbA*^+^ control (heatmaps of Fig. 5a,b), except in the cases of C’*hmbA-gfp* strain on allantoin (increased *ER*, *P* < 0.05) and C’*hmbA*^+^ strain on uric acid as sole nitrogen-source (decreased *ER*, *P* < 0.05). This indicates that Nhp6Ap and HmbA complementation resulted in a similar phenotype as *hmbA*^+^ in all carbon-sources and most nitrogen sources.

Severe heat stress (42 °C) has a more adverse effect on the growth of *hmbAΔ* than that of the *hmbA*^+^ strain (left panel of Fig. 5c), which is also confirmed by a significantly decreased *ER* (*P* < 0.0001). Although *hmbA* and *NHP6A* complemented the *hmbA* deletion phenotypes on all carbon- and most of the nitrogen-sources (right panel of Fig. 5a,b), the heat sensitivity was not reversed to the wild-type-like level in C’*hmbA*^+^ and C’*NHP6A* strains as shown by significantly reduced *ER* values (right panel of Fig. 5c). Complementation of *hmbAΔ* by *hmbA-gfp* (C'*hmbA-gfp*) reversed the heat sensitivity to the wild-type level (right panel of Fig. 5c, Supplementary Fig. S1*e*). Supplementation of the medium with 1 M sorbitol remediated the heat sensitivity at 42 °C in all tested strains (Fig. 5c, Supplementary Fig. S1*e*). While sorbitol acts as osmotic stabilizer when used in 1 M concentration, increasing the concentration to 2 M causes an osmotic stress. At 37 °C, the *hmbAΔ* strain tolerated the osmotic stress on 2 M sorbitol better (increased *ER* value, *P* < 0.01) than the *hmbA*^+^ or the complemented controls did.

Finally, we tested several other stress responses. The *hmbAΔ* strain was more sensitive to the oxidative stress agent menadione (at 50 µM) than the *hmbA*^+^ strain (decreased *ER* value*, P* < 0.05) (Fig. 5d, Supplementary Fig. S1f). Cell wall disruptors (10 µM Calcofluor White, 50 µM Congo Red, 35 µM SDS) were better tolerated by *hmbAΔ* than by *hmbA*^+^ (increased *ER* values, *P* < 0.05, *P* < 0.0001 and *P* < 0.01, respectively), however *hmbAΔ* was more sensitive to 1 mM caffeine than *hmbA*^+^ (decreased *ER* value, *P* < 0.0001) (Fig. 5d, Supplementary Fig. S1f). The differential responses of *hmbAΔ* and *hmbA*^+^ in the presence of cell wall disruptors might reflect strain-specific differences in the cell wall architecture. The supplementation of the media with 1 M sorbitol resulted in an increased *ER* value in all tested strains (Fig. 5d, Supplementary Fig. S1f).

Response to the presence of a heavy metal stressor (50 µM cadmium sulphate) in *hmbAΔ* was similar to that of *hmbA*^+^ and the C'*hmbA* and C’*hmbA-gfp* complemented strains (Supplementary Fig. S1f). Notably, the C’*NHP6A* strain showed extremely negative response to cadmium sulphate, which was rescued by the supplementation of the medium with 1 M sorbitol (Supplementary Fig. S1f).

To summarise, the above described growth tests all indicate that HmbA has a large contribution in the normal response to various environmental changes.

### Deletion of *hmbA* results in altered chitin deposition at the hyphal tips, production of thin hyphae and an abnormal mode of hypha elongation

**Figure 6:**
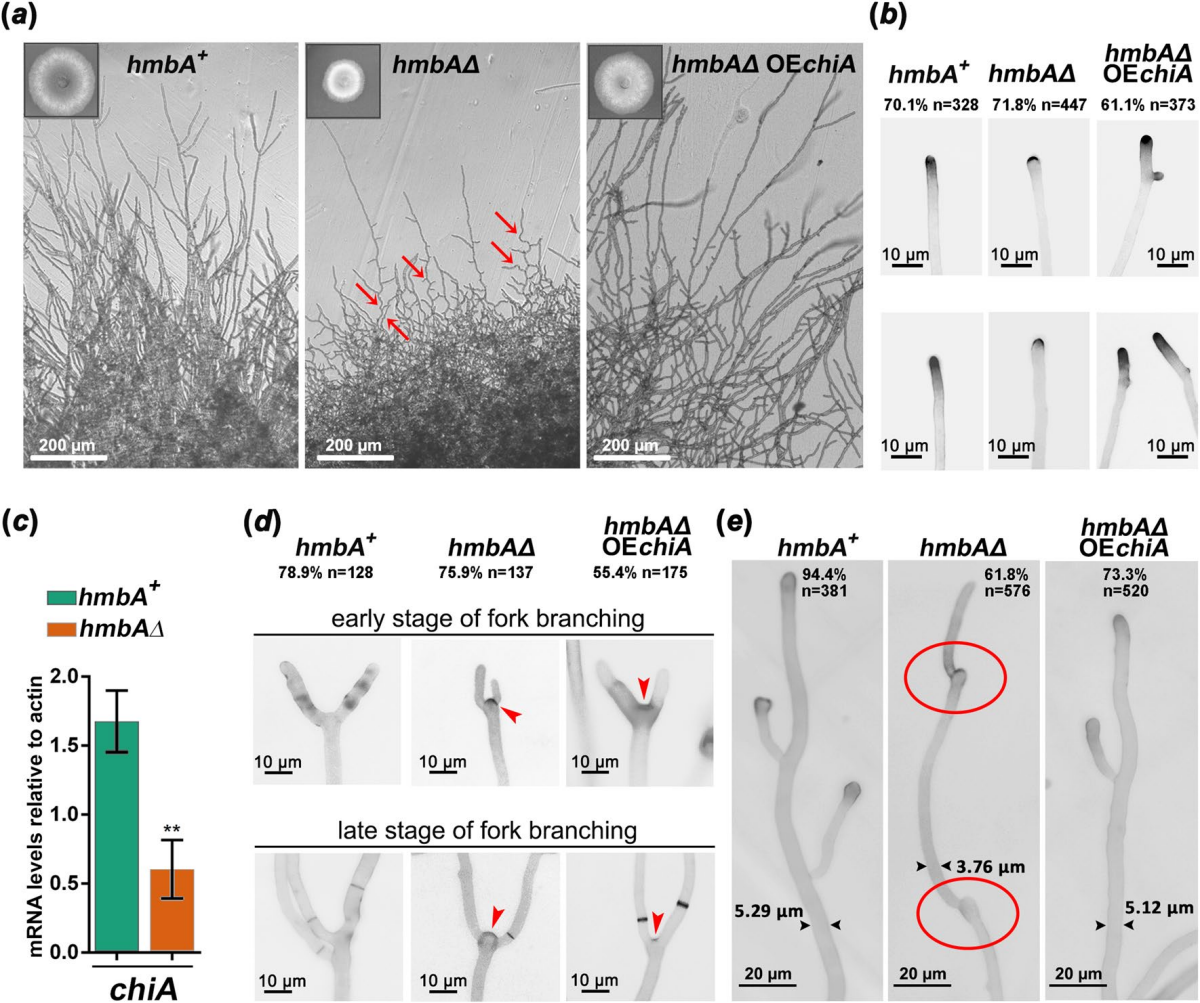
Downregulation of *chiA* altered morphology and chitin distribution in *hmbA∆*. (**a**) The images show the morphology of elongated hyphae across three tested strains. Bright field images were documented with Leica DMI 4000B, DFC295 detector. Scale bars are shown. Red arrows denote zig-zag shaped hyphae. Insets show colonies grown on glucose-nitrate minimal medium at 37 °C for 3 days. (**b**) The images show Calcofluor White staining of chitin at the hyphal tips across three tested strains. Scale bars are shown. Microscopy was carried out with Zeiss Axioobserver 7 Axiocam 503 mono detector with DAPI filter setting. (**c**) The figure shows the mRNA levels of *chiA* in the tested strains measured by RT-qPCR. Strains were grown on glucose-nitrate minimal medium at 37 °C for 9 h. RT-qPCR data were processed according to the standard curve method ^44^ with γ-actin transcript (*actA*/AN6542) as reference. Standard deviations of three independent experiments are shown. Primers are listed in Supplementary Table S4. (**d**) The images show the fork-branching at the hyphal tips of the tested strains at early and later stages of fork-branching. Arrowheads denote the relicts of hyphal tips that underwent fork-branching. Scale bars are shown. (**e**) The images show hypha elongation across three tested strains. Samples were stained with Calcofluor White. Scale bars are shown. Red circles in midsections of elongated hyphae mark the relicts of arrested hyphal tips that underwent re-initation of polar growth to continue the hypha elongation. This non-canonical way of elongation resulted in zig-zag shaped hyphae (denoted by red arrows in panel *a*). Tested strains were *hmbA*^+^ (*hmbA*^+^ control, HZS.120), *hmbA∆* (*hmbA* deletion strain, HZS.320) and *hmbA∆* OE*chiA* (*chiA* overexpressing *hmbAΔ* strain, HZS.921). The complete genotypes are listed in Supplementary Table S2. Frequency of occurrence of the presented phenotypes are indicated at the top of the images in panels *b*, *d* and *e*.

To further characterize *hmbAΔ*, we focused on several morphological features of the hyphae. The decrease in hyphal elongation rate described above is observed in all tested media (Fig. 5). The slow growth was accompanied by compact mycelium formation, composed from thin and aberrantly running (zig-zag shaped) hyphae (Fig. 6a,e). Most (61.81%, n = 576) of the *hmbAΔ* hyphae run in a markedly wavy course and have more side-branches than the *hmbA*^+^ control (Fig. 6a). The C’*hmbA-gfp* and C’*hmbA* complemented strains showed wild-type morphology, while the recovery was only partial in the C’*NHP6A* complemented strain (Supplementary Fig. S2*a*).

Growth tests on media supplemented with cell wall disruptor agents indicated that the cell wall architecture may differ between the *hmbAΔ* and *hmbA*^+^ strains (Fig. 5d), therefore, Calcofluor White staining was used to monitor the distribution of chitin at the hyphal tips. Chitin staining was intense at the top edge of a large fraction (70.1%, n = 328) of the wild type hyphae and gradually decreased in the sub-apical area (Fig. 6b, Supplementary Fig. S1*b*). Such a wild-type like diffuse chitin distribution in the sub-apical area was observed only in a small fraction (28.2%, n = 447) of the *hmbAΔ* hypha tips (Fig. 6b, Supplementary Fig. S1*b*), which indicates that remodelling of cell wall at the subtending zone below the apex is damaged in *hmbAΔ*.

Remodelling of the cell wall, especially at the subtending zone, requires the action of endochitinases and endoglucanases^19^ (recently reviewed in^20,21^). We found that *chiA* (codes for endochitinase supposedly involved in cell wall remodelling^22^) is significantly downregulated in the *hmbAΔ* mutant by 2.77 fold (*P* < 0.01) as compared to the *hmbA*^+^ control (Fig. 6c). To test whether *chiA* downregulation contributes to the aberrant hyphal growth phenotype, we overexpressed the *chiA* gene in an *hmbAΔ* strain (*hmbAΔ* OE*chiA*). Overexpression of *chiA* resulted in a normal (wild-type) hypha elongation pattern (the colony size became wild-type-like), improved the hypha morphology of elongated hyphae (73.26% of them were wild-type like, n = 520) and restored the diffuse distribution of chitin in the sub-apical area (61.13% was wild-type like, n = 373) supporting the notion that the normal expression of *chiA* might be critical for the above processes (Fig. 6a,b). The frequencies of occurrence of wild-type like and deletion-like hypha characteristics (hypha tips, fork-branches and shape of elongated hyphae) in *hmbAΔ* OE*chiA* strain fell between the values measured in *hmbA*^+^ control and *hmbAΔ*, revealing the common role of *chiA* in these related phenotypic traits (Supplementary Fig. S2*b*).

Notably, chitin deposition is unusually strong in a large proportion (75.9%, n = 137) of the nascent branching sites at the tips (fork-branching sites) in *hmbAΔ* (Fig. 6d and more in Supplementary Fig. S2*c*) compared to the *hmbA*^+^ control (21.1%, n = 128). This abnormality was not resolved with aging, as the deposited chitin seems to be permanently present at the base of the aged fork-branches (see late-stage of fork-branched hyphae in Fig. 6d). Overexpression of *chiA* in the *hmbAΔ* strain restored the phenotype of fork-branches to normal in 44.57% (n = 175) of fork-branches (Fig. 6d, Supplementary Fig. S2*b*). Because a *chiA* deletion on its own does not result in abnormal polarized growth^23^, we hypothesise that additional cell-wall remodelling genes are also misregulated in *hmbAΔ*.

We thus outline the following scenario that might lead to the above described phenotypes of *hmbAΔ*. Downregulation of cell wall remodelling factors (such as endochitinase *chiA*) leads to incomplete cell wall remodelling at the subtending zone below the apex. This might result in transient arrest of hypha elongation accompanied by the enlargement of apical ends and chitin accumulation at the perimeter of the enlarged tips, without showing clear signs of diffuse presence of chitin in the subapical region. Inadequate cell wall remodelling might hinder tip elongation, which, however, might be re-initiated in the arrested state by a presumably non-canonical process (similar to that operates in conidiospores during germ tube formation). A support for this scenario is our observation that abnormal chitin-rich round structures are present at the mid-sections of the *hmbAΔ* hypha causing zig-zag shaped hyphae (Fig. 6e and Supplementary Fig. S2*c*). Future work should decipher the molecular events that lead to such re-initiation of elongation, specifically to whether it is dependent on the same machinery that maintains the already established polar growth or it involves an alternative pathway.

### Germination of *hmbAΔ* conidiospores is delayed

**Figure 7:**
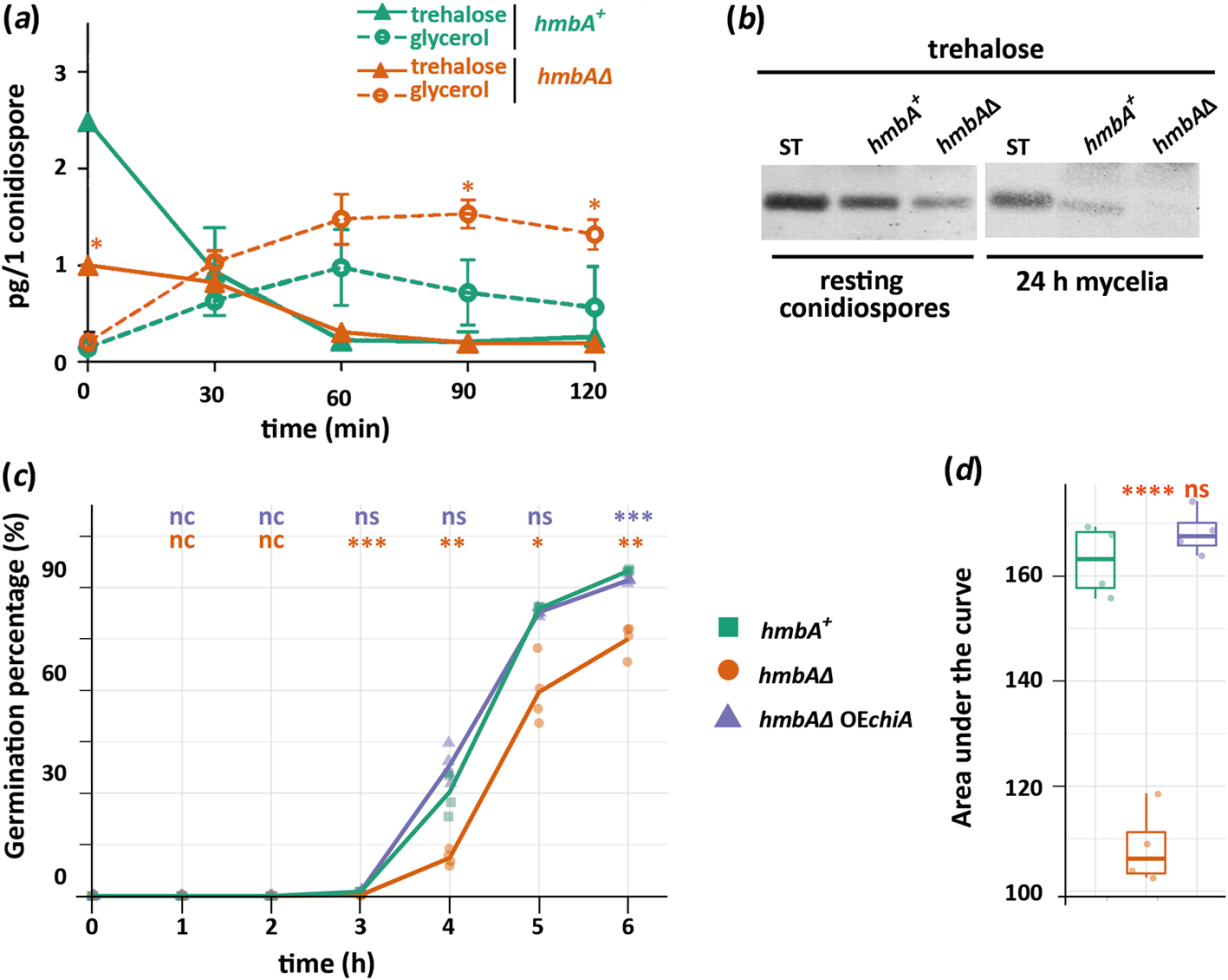
Comparison of trehalose content and metabolism of trehalose to glycerol in *hmbAΔ* and *hmbA*^+^ conidiospores and characterization of conidiospore germination. (**a**) HPLC-detected conversion of trehalose to glycerol during the first two hours of germination in *hmbAΔ* and *hmbA*^+^ conidiospores. Measurements started with the analysis of resting conidiospores at the 0 h time point. Colour and figure codes are shown in the figure legend. Mean of three biological replicates are shown. Error bars represent standard deviation. Significant differences (Student’s *t*-test) are marked with asterisks (* *P* < 0.05); (**b**) Detection of trehalose by TLC in conidiospores and mycelia of *hmbAΔ* and *hmbA*^+^ strains. ST indicates trehalose standard. (**c**) Characterization of conidiospore germination rate in *hmbA*^+^, *hmbAΔ* and *hmbAΔ* OE*chiA* strains. 10^3^ conidiospores were inoculated in cell culture dishes with glass bottom and incubated on 37 °C. Germination was monitored and documented by using Zeiss Axioobserver 7 microscope with Axiocam 503 mono detector. Germination percentage was calculated by normalizing the number of germinated conidiospores with the number of the non-germinated conidiospores. Solid lines denote the mean of four biological replicates. Colour and figure codes are shown in the legend embedded in the figure. Significant differences (Student’s *t*-test) are marked with asterisks (* *P* < 0.05; ** *P* < 0.01; *** *P* < 0.001; nc: non-calculated due to each measured value is zero, ns: non-significant). Significance denoted by orange and blue corresponds to pairwise comparison of the control *versus hmbAΔ* and control *versus hmbAΔ* OE*chiA* strain, respectively. (**d**) The figure shows the area under the curves presented in the germination rate graph (panel *c*) based on four biological replicates. Box plots show the median, first and third quartiles, with whiskers showing the 5th and 95th percentiles. Significant changes (Student’s *t*-test) are marked with asterisks **** *P* < 0.0001; ns: non-significant). Used strains were *hmbA*^+^ as control (HZS.120); *hmbAΔ* (*hmbA* deletion strain; HZS.320) and *hmbAΔ* OE*chiA* (*chiA* overexpressing *hmbAΔ*; HZS.921). The complete genotypes are listed in Supplementary Table S2. The original TLC images are presented in Supplementary Fig. S4*b*.

We also investigated conidiospore germination in *hmbAΔ* strain. Deletion of *hmbA* resulted in a 30 min delay in the germination of conidiospores (Fig. 7c). Conversion of the trehalose deposited in resting conidiospores to glycerol is necessary for the swelling of conidiospores during the isotropic growth phase of germination^24^. According to this, we hypothesized that the delay of germination of *hmbAΔ* conidiospores might be associated with impaired trehalose content or impaired conversion of trehalose to glycerol. By measuring the trehalose and glycerol content of *hmbA*^+^ and *hmbAΔ* in the first 120 min of germination (isotropic growth phase) by using HPLC, we found that, despite the fact that the trehalose content of *hmbAΔ* resting conidiospores was significantly lower (*P* < 0.05) compared to that of *hmbA*^+^, trehalose in *hmbAΔ* was metabolised at the same rate as in *hmbA*^+^ after 30 min of incubation (Fig. 7a). Remarkably, the lower initial trehalose content of *hmbAΔ* conidiospores did not limit the glycerol production, moreover, significantly more (*P* < 0.05) glycerol was produced in the *hmbAΔ* conidiospores (Fig. 7a). Besides trehalose, various polyols (such as mannitol) could also serve as a source of glycerol during conidiospore germination ^25,26^ that might explain the high level of glycerol in swelled *hmbAΔ* conidiospores.

TLC detection of trehalose content further confirmed the lower trehalose content of *hmbAΔ* conidiospores compared to that of *hmbA*^+^ (Fig. 7b). Similar quantitative differences in trehalose content were also observed in mycelia (Fig. 7b).

Since the level of glycerol is normal in the *hmbAΔ* conidiospores during the isotropic growth phase, the germination delay is not caused by a failure of swelling. After swelling, an area of the conidiospore cell wall must undergo a remodelling process where the germ tube emerges and polar growth is initiated. It is reasonable to assume that any impairment of cell wall remodelling might affect germination. The endochitinase ChiA was found to be required for normal germination by acting as a cell wall remodeller in conidiospores as deletion of *chiA* results in delayed germination as well as decreased colony growth^22^. This is consistent with the impaired growth phenotype of *hmbAΔ* strain, in which the *chiA* expression is downregulated (Fig. 6c), overexpression of *chiA* rescued the impaired growth phenotype of *hmbAΔ* (Fig. 6a–e) and restored the germination dynamics to the *hmbA*^+^ level (Fig. 7c,d).

### HmbA regulates the production of the secondary metabolite sterigmatocystin in a nitrogen source-dependent way

**Figure 8:**
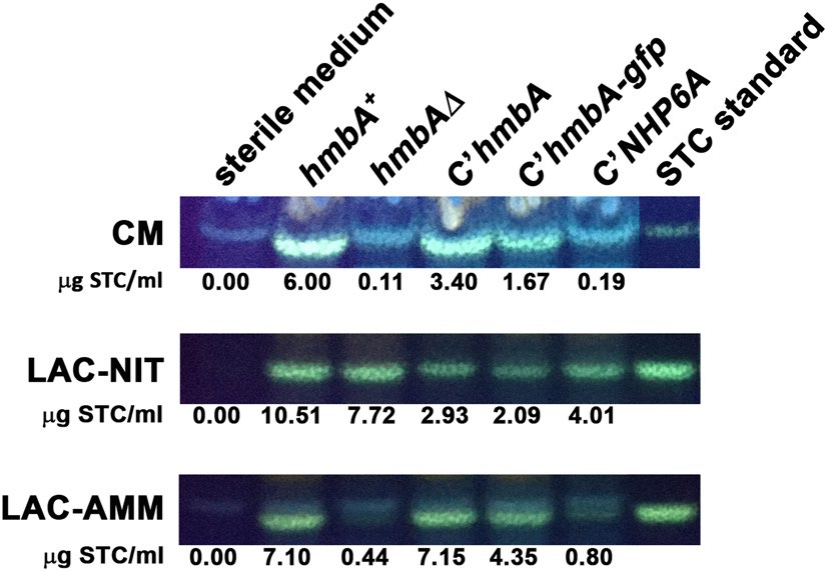
Sterigmatocystin production on various media in *hmbA*^+^ control, *hmbAΔ* and various complemented *hmbAΔ* strains detected by TLC and quantified by HPLC. CM: complete medium, LAC-NIT: minimal medium with lactose carbon-source and sodium-nitrate nitrogen-source; LAC-AMM: minimal medium with lactose carbon-source and diammonium L-(+)-tartrate nitrogen-source. Used strains were *hmbA*^+^ as control (HZS.120); *hmbAΔ* (*hmbA* deletion strain, HZS.320); *C’hmbA* (*hmbAΔ* complemented with *hmbA*, HZS.621), C’*hmbA-gfp* (*hmbAΔ* complemented with *hmbA-gfp*, HZS.371); C’*NHP6A* (*hmbAΔ* complemented with *NHP6A*, HZS.834). Sterile medium was used as negative control. STC is sterigmatocystin standard (Sigma). The complete genotypes are listed in Supplementary Table S2. The original TLC images are presented in Supplementary Fig. S4*c*.

To investigate the role of HmbA in the production of the widely studied secondary metabolite, sterigmatocystin (STC), we performed quantification of STC by using TLC and HPLC methods. STC production was barely detectable in *hmbAΔ* on complete medium and on lactose—ammonium minimal medium compared to the *hmbA*^+^ control (Fig. 8). However, the produced STC was comparable to that observed in *hmbA*^+^ on lactose—nitrate minimal medium (Fig. 8) suggesting that HmbA is involved in the regulation of STC production in a nitrogen-source-dependent manner. The mechanism underlying this phenomenon is yet-to-be explored. Notably, even though expression of *hmbA* and *hmbA-gfp* in *hmbAΔ* strain re-established the *hmbA*^+^-like STC production, the heterologous expression of *NHP6A* did not improve the STC production in *hmbAΔ* (Fig. 8).

## Conclusion

HmbA is structurally very similar to the HMGB1-like Nhp6p of *S. cerevisiae*. The DNA binding surface of HmbA is remarkably identical with that of Nhp6p, which may explain why the Nhp6Ap is interchangeable with HmbA, at least in those functional roles where the HMG-B protein’s role is primarily dependent on its DNA-binding properties (such as *SNR6* expression in *S. cerevisiae*^12,13^). HmbA is also interchangeable with Nhp6Ap, most of the physiological functions under investigation were restored by Nhp6p in the *hmbAΔ* strain (ascospore formation in the fruiting bodies, response to change of carbon- and nitrogen-sources, radial growth rate, hypha elongation by mostly wild-type like polarized growth). We hypothesize that the physiological functions of HmbA, which are restored by the heterologous expression of *NHP6A* in an *hmbAΔ* strain predominantly depend on the DNA binding ability of the HmbA protein. It would be interesting to investigate whether those HmbA functions, which were not restored by Nhp6Ap (production of STC, response to menadione and cadmium-sulphate generated oxidative and heavy metal stress, respectively) depend on the interaction of HmbA with specific, yet unexplored protein elements of the chromatin.

In this work we propose a connection between the slow growth rate, the abnormal mode of polarized growth maintenance and the regulatory role of HmbA, that is highly dependent on the proper expression of cell wall re-modeller *chiA.* Besides *chiA*, HmbA presumably has an impact on the expression of other cell wall re-modeller genes and thereby the altered polarized growth of *hmbAΔ* might be the overall effect of the inadequate expression of numerous wall-re-modellers. By using phenotypic characterization of the *hmbA* deletion phenotype, our work shed light on the pivotal role of endochitinases in cell wall remodelling and the normal mode of maintenance of polar growth and, as such, our novel results complete previous work, which has failed to demonstrate a connection between hyphal polar growth and the level of endochitinases in either *A. nidulans* or *A. fumigatus*^22,23,27^.

## Methods

### Strains, cultures, growth conditions

The *A. nidulans* and *S. cerevisiae* strains used in this study are listed in the Supplementary Table S2. Standard *Aspergillus* genetic markers, complete medium (CM) and minimal medium (MM) are described at the following URL: http://www.fgsc.net/Aspergillus/gene_list/. Media were supplemented with vitamins (www.fgsc.net) according to the requirements of each auxotrophic strain. The various stressors, carbon- and nitrogen-sources used in the experiments are listed in Supplementary Table S3. When indicated, media were supplemented with 1 M sorbitol as osmotic stabilizer. Media used for *S. cerevisiae* work are described by Tong and Boone^28^. Supplementations are detailed in the relevant method sections.

### Inoculation from pre-grown mycelia

As the *hmbAΔ* conidiospores showed a delay germination compard to the *hmbA*^+^ control, we started the growth tests by inoculating pre-grown, non-sporulating mycelial discs instead of conidiospores as previously described^29^.

### Construction of *S. cerevisiae nhp6AΔBΔ* double deletion mutant

To generate a double deletion strain for *NHP6A* and *NHP6B,* first we obtained the *nhp6aΔ::KanMX* and the *nhp6bΔ::NatMX* strains from the YKO Mat-a collection^30^ and SGA (Synthetic Genetic Array) query collection 28, respectively. The KanMX deletion cassette of the *nhp6aΔ* strain was swapped to HphMX marker (see Supplementary Methods). We generated the double mutant *nhp6AΔBΔ* by genetic cross of *nhp6aΔ::HphMX* with *nhp6bΔ::NatMX* following an established protocol^28^ (see Supplementary methods). Gene deletion was checked by PCR (see Supplementary methods). Primers are listed in Supplementary Table S4.

### Construction of *hmbA* expressing *nhp6AΔBΔ S. cerevisiae* strain

cDNA of *A. nidulans hmbA* gene was cloned into the *S. cerevisiae* expression vector, M4801 (http://www.addgene.org/51664/) followed by the truncation of the P_*GAL*_ promoter and cloning of the promoter sequence of *NHP6A* (P_*NHP6A*_). For details see Supplementary Methods. The resulted vector (M4801-P_*NHP6A*_-C'*hmbA*) (Supplementary Fig. S3*a*) was transformed into *nhp6AΔBΔ* strain (HZS.891). Selection and analysis of the transformant strains are described in Supplementary Methods. Used primers are listed in Supplementary Table S4.

### Construction of *NHP6A*, *hmbA-gfp* and *chiA* expressing *hmbAΔ* strains

Transformation cassettes were constructed by using double-join PCR (DJ-PCR) method^31^ and cloning. In order to obtain *NHP6A* complemented *hmbAΔ* strain (C'*NHP6A*) pAN-HZS-19 expression vector was constructed (construction is described in Supplementary methods) (Supplementary Fig. S3*b*). The vector carries the coding sequence of *NHP6A* from *S. cerevisiae* driven by the physiological promoter of *hmbA*.

In order to obtain the *hmbA-gfp* expressing strain (C'*hmbA-gfp*), a gene-substitution cassette was composed from the *hmbA-gfp* gene phusion, termination sequence of *trpC* gene and the *pantoB*^+^ selection marker gene flanked by the genomic regions upstream and downstream to *hmbA* (construction is described in Supplementary methods).

In order to obtain *chiA* overexpressing *hmbAΔ* strain (*hmbAΔ* OE*chiA*), the coding sequence of *chiA* gene was amplified and cloned into pAN-HZS-1 vector^32^ resulting in pAN-HZS-31 overexpression vector (Supplementary Fig. S3*b*) (construction is described in Supplementary methods).

The resulted constructions (pAN-HZS-19, the *hmbA-gfp* cassette and pAN-HZS-31) were transformed into an *hmbAΔ* strain (HZS.320) and pantothenic acid prototroph transformant strains were collected. The copy number was calculated as described by Herrera et al.^33^ (detailed in Supplementary methods). The verified transformants C'*hmbA-gfp* (HZS.371, with two copies of *hmbA-gfp*), C’*NHP6A* (HZS.834, with one copy of *NHP6A*) and *hmbA∆* OE*chiA* (HZS.921, with one copy of *chiA*) were used in the experiments. Complete genotypes of the strains and all the used primers are listed in Supplementary Tables S2 and S4, respectively.

### Microscopy

For the study of HmbA localization, 10^4^ conidiospores of C’*hmbA-gfp* (HZS.371) was germinated for 5.5 h at 37 °C on the surface of cover slips submerged in liquid MM (GLU-NIT). Nuclei were stained with DAPI (4′,6-diamidino-2-phenylindole) (Sigma) according to May^34^. Germlings were studied in Zeiss Axiolab A fluorescent microscope using Zeiss filter set 15 (GFP) and 49 (DAPI). Chitin staining was carried out by incubating young mycelia grown over cellophane^35,36^ in 10 µg/ml CFW stain (Fluka) dissolved in PBS (phosphate buffered saline) for 10 min followed by 3 × wash in PBS and microscopy with Zeiss Axioobserver 7 Axiocam 503 mono detector with DAPI filter setting.

### DNA manipulation

Total DNA was prepared from *A. nidulans* as described by Specht et al.^37^. Transformations of *A. nidulans* protoplasts were performed as described by Antal et al.^38^. The protoplasts were prepared from mycelia grown over cellophane^35,36^ by using a 4% solution of Glucanex (Novozymes, Switzerland) in 0.7 M KCl solution. Transformation of 5 × 10^7^ protoplasts was carried out with 100–1 µg of fusion PCR products or with 1–5 µg of plasmids. Transformation of *S. cerevisiae* was carried out with the lithium acetate method using single-stranded carrier DNA^39^. Genomic DNA of *S. cerevisiae* was prepared as described by Bodi et al.^40^. For cloning procedures, *E. coli* JM109^41^ was used and transformation of *E. coli* was performed according to Hanahan^42^. Plasmid extraction from *E. coli* and other DNA manipulations were done as described by Sambrook et al.^43^.

### RNA work

For the purpose of Northern analysis, total RNA was isolated from various developmental stages of *A. nidulans* (from 1 to 6 h) by a standard protocol using the Trizol Reagent of Invitrogen (CA, USA). Northern blot analysis was performed using the glyoxal method^43^. Equal RNA loading was calculated by optical density measurements (260/280 nm). [^32^P]-dCTP labelled *hmbA* and *18S rRNA* gene-specific DNA molecules were used as gene probes using the random hexanucleotide-primer kit following the supplier’s instructions (Promega). As a loading control, the expression of *18S rRNA* gene was detected. For the purpose of mRNA analysis by reverse transcription qPCR (RT-qPCR), total RNA was isolated from exponentially growing *S. cerevisiae* cultures (OD_600_ = 0.3–1) using RiboPure-Yeast (Invitrogen) kit and from *A. nidulans* mycelia after 9 h incubation using a NucleoSpin RNA Plant kit (Macherey–Nagel) and RNase-Free DNase (Qiagen) according to the manufacturers’ instructions. RNA quality was assessed by using agarose gel electrophoresis. DNA contamination of the RNA samples was checked by performing qPCR on 1 µg RNA samples with *A. nidulans* γ-actin coding gene (*actA*/AN6542) or *S. cerevisiae UBC6* (ubiquitin-conjugating enzyme coding gene) specific primers. Samples showing higher than 32 cycle Cq values in the DNA contamination test were used for reverse transcription. cDNA synthesis was carried out with a mixture of oligo-dT and random primers using a RevertAid First Strand cDNA Synthesis kit (Fermentas). RT-qPCR was carried out in a CFX96 Real-Time PCR System (Bio-Rad) with Maxima SYBR Green/Fluorescein qPCR Master Mix (Fermentas) reaction mixture (94 °C for 3 min followed by 40 cycles of 94 °C for 15 s and 60 °C for 1 min). The data processing was performed by the standard curve method^44^. The gene expression values of genes of interest were normalized to *actA* or *UBC6* reference genes. All the used primers are listed in Supplementary Table S4.

### Extraction and detection of STC by TLC and HPLC

TLC was carried out on chloroform extracts of 6-day-old *A. nidulans* colonies using toluol : ethylacetate : formic acid (50:40:10 V/V/V) for the development of the chromatogram^45^ (for details see Supplementary Methods). HPLC detection of STC was carried out using a Shimadzu HPLC system (Shimadzu, Kyoto, Japan) and Purosphere Star RP18e, 250 × 4 mm, 5 µm column (Merck KGaA, Darmstadt, Germany) at a column temperature of 40 °C. Details are described in the Supplementary Methods.

### Extraction and detection of trehalose and glycerol by TLC and HPLC

For the monitoring of metabolism of conidial trehalose to glycerol during the swelling of conidiospores, sugars and polyalcohols were extracted from 10^9^ conidiospores after 0, 30, 60, 90, 120 min and 24 h of incubation by using 5% (m/V) trichloroacetic acid (TCA) as described in the Supplementary methods. TLC chromatogram of trehalose was developed in chloroform : methanol : distilled water : acetic acid (55:33:8:1 V/V/V/V) (for details see Supplementary methods).

TCA extracted samples were analysed in separated chromatographic runs for both glycerol and trehalose contents by using the Shimadzu HPLC system as described above in the STC analysis section (Shimadzu, Kyoto, Japan) except that the original detector was replaced with a refractive index detector (RID-10). For details see the Supplementary Methods.

### Protein modelling

Initial protein models were obtained by I-Tasser^46^, whereas the refined models were obtained by ModRefiner^47^. The generated pdb files were further analysed in UCSF Chimera 1.14. Ramachandran plot analysis was carried out by using Procheck server (https://servicesn.mbi.ucla.edu/PROCHECK/). Electrostatic potential was calculated with Delphi Web Server with the default parameters (http://compbio.clemson.edu/sapp/delphi_webserver/).^48,49^ The resulted pqr file was submitted to Chimera and electrostatic surface colouring was done by the Coulombic Surface Colouring application of Chimera.

### *S. cerevisiae* fitness measurements

Growth was assayed by monitoring the optical density at 600 nm (OD_600_ value) of liquid cultures by using 384 well density microtiter plates following a previous protocol^50^. The growth curves were monitored over a 72 h incubation period in a Powerwave HT plate reader (BioTek Instruments Inc). During the kinetic run, the optical density of each well was recorded at 600 nm (OD_600_) every 5 min. Between the optical readings, the cultures were incubated at 30 °C, with alternating shaking speed (1000–1200 rpm). A modified version of a published procedure^51,52^, implemented in R^53^ was used to estimate several fitness components, including growth rate, doubling time, optical density increment, and length of the lag phase.

## Supplementary Information

Supplementary Information 1.

Supplementary Information 2.

## Data Availability

All experimental data are shown in either the main text or in the Supplementary files (Supplementary Information File and Supplementary Dataset File). Sequences used in the study were downloaded from public databases (Nhp6Ap from https://www.yeastgenome.org/locus/S000006256#protein; HmbA from https://fungidb.org/fungidb/app/record/gene/AN2885).
