# Supplementary material for: The role of the Aspergillus nidulans high mobility group B protein HmbA, the orthologue of Saccharomyces cerevisiae Nhp6p
Source: Sci Rep. 2022 Oct 15;12:17334. doi: 10.1038/s41598-022-22202-3 (PMC9569327; doi:10.1038/s41598-022-22202-3)
Supplement: Supplementary file 2 — Supplementary Information 2. [file 41598_2022_22202_MOESM2_ESM.pdf]

SUPPLEMENTARY INFORMATION

for

The role of the *Aspergillus nidulans* High Mobility Group B protein HmbA, the orthologue of *Saccharomyces cerevisiae* Nhp6p

Judit Ámon[1+], Gabriella Varga[1+], Ilona Pfeiffer[1], Zoltán Farkas[2], Zoltán Karácsony[1#], Zsófia Hegedűs[1], Csaba Vágvölgyi[1], and Zsuzsanna Hamari[1*]

[1]University of Szeged Faculty of Science and Informatics, Department of Microbiology, Szeged, Hungary

[2]Synthetic and Systems Biology Unit, Institute of Biochemistry, Biological Research Centre, Eötvös Loránd Research Network, Szeged, Hungary

*Corresponding author:

Zsuzsanna Hamari

hamari@bio.u-szeged.hu

Content:

Supplementary Figures: Figs. S1-S4

Supplementary Tables: Tables S1-S4

Supplementary Methods

SUPPLEMENTARY FIGURES

for

The role of the *Aspergillus nidulans* High Mobility Group B protein HmbA, the orthologue of *Saccharomyces cerevisiae* Nhp6p

by

Judit Ámon[1+], Gabriella Varga[1+], Ilona Pfeiffer[1], Zoltán Farkas[2], Zoltán Karácsony[1#], Zsófia Hegedűs[1], Csaba Vágvölgyi[1], and Zsuzsanna Hamari[1*]

[1]University of Szeged Faculty of Science and Informatics, Department of Microbiology, Szeged, Hungary

[2]Synthetic and Systems Biology Unit, Institute of Biochemistry, Biological Research Centre, Eötvös Loránd Research Network, Szeged, Hungary

*Corresponding author:
Zsuzsanna Hamari
hamari@bio.u-szeged.hu

Content:

Supplementary Figure S1: Response of *hmbAΔ* and the complemented strains to various environmental conditions compared to a control condition.

Supplementary Figure S2: Presentation of micromorphology in *hmbA*$^{+}$ control, *hmbAΔ* and in the various complemented and *chiA* overexpressing strains.

Supplementary Figure S3: Schematic presentation of the vectors constructed in this work.

Supplementary Figure S4: Original images of Northern blots and TLC plates presented in Figures 4a, 7b and 8.

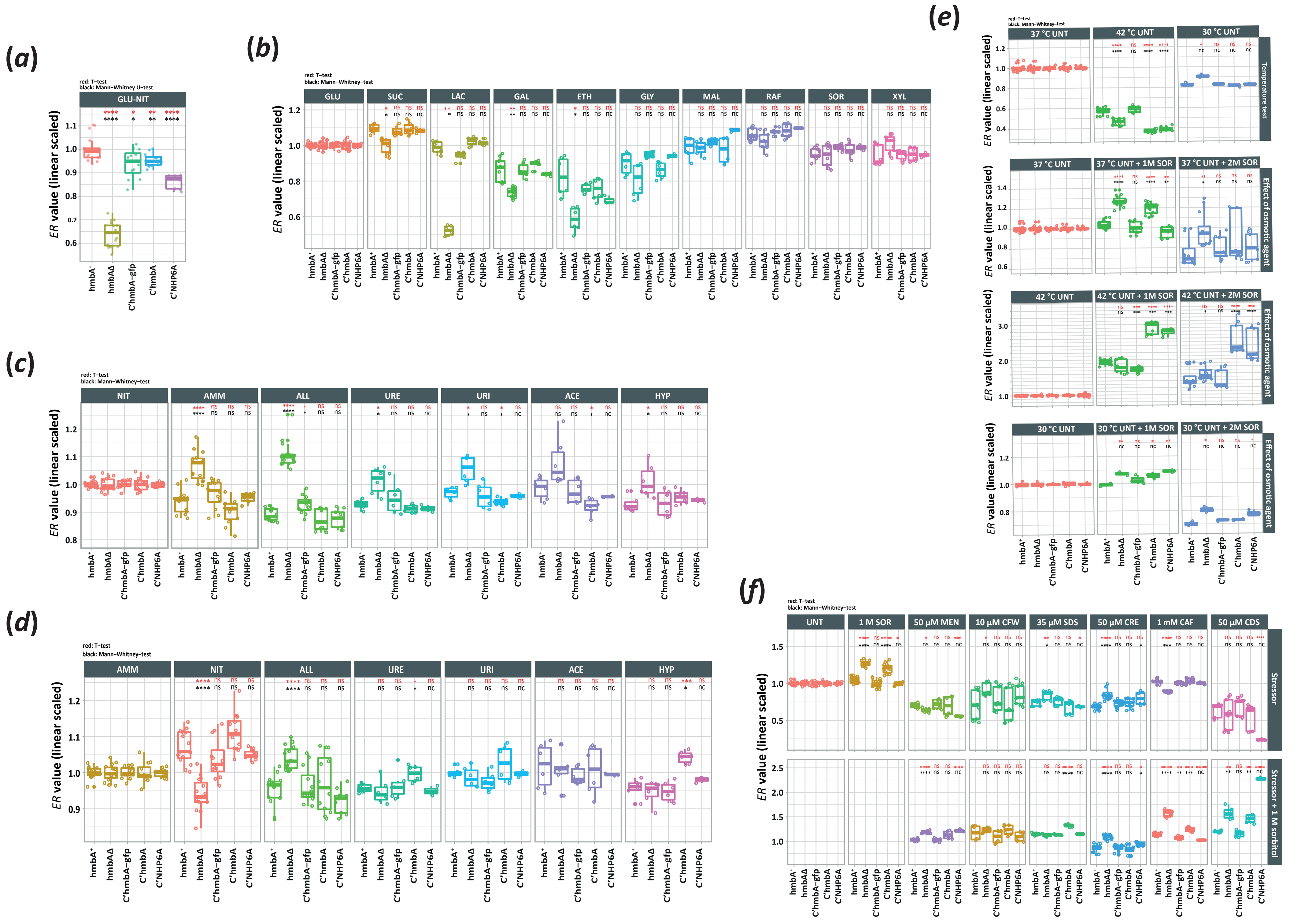

(a)
red: T-test
black: Mann-Whitney U-test
GLU-NIT
ER value (linear scaled)
hmbA+
hmbAΔ
C'hmbA-gfp
C'hmbA
C'NHP6A
(b)
red: T-test
black: Mann-Whitney-test
GLU
SUC
LAC
GAL
ETH
GLY
MAL
RAF
SOR
XYL
(c)
NIT
AMM
ALL
URE
URI
ACE
HYP
(d)
AMM
NIT
ALL
URE
URI
ACE
HYP
(e)
37 °C UNT
42 °C UNT
30 °C UNT
Temperature test
37 °C UNT + 1M SOR
37 °C UNT + 2M SOR
Effect of osmotic agent
42 °C UNT + 1M SOR
42 °C UNT + 2M SOR
30 °C UNT + 1M SOR
30 °C UNT + 2M SOR
(f)
UNT
1 M SOR
50 μM MEN
10 μM CFW
35 μM SDS
50 μM CRE
1 mM CAF
50 μM CDS
Stressor
Stressor + 1 M sorbitol

Supplementary Figure S1. Response of *hmbAΔ* and the complemented strains to various environmental conditions compared to a control condition.

The boxplot shows the environmental response (*ER* value) of all examined strains to the tested conditions compared to the control condition. The *ER* value was calculated strainwisely by using the following formula: $size_{tested\ condition}$/average $size_{control\ condition}$, where $size_{tested\ condition}$ corresponds to the colony size of a given strain in a tested condition, and average $size_{control\ condition}$ corresponds to the average size of a given strain measured in the corresponding control condition (calculated data are presented together with all raw data in Supplementary Dataset File). During the calculation of the *ER* values, the environmental condition displayed in the first subpanel was used. The experiments were executed at least in three biological replicates. The calculations were done separately on each biological replicate and the calculated *ER* values were plotted by the ggplot2 package of R [1]. Box plots show the median, first and third quartiles, with whiskers showing the 5th and 95th percentiles. To assess significant differences between the *ER* values of $hmbA^{+}$ control and the tested strains (panel *a*) or significant differences in the *ER* values of *hmbAΔ* and in the various reconstituted strains to that of the $hmbA^{+}$ control (panels *b*-*f*), we used Student's *t*-test (red asterisks/letters) and Mann-Whitney U-test (black asterisks/letters): * $P < 0.05$; ** $P < 0.01$; *** $P < 0.001$; **** $P < 0.0001$; nc: non-calculated due to low sample size; ns: non-significant.

(a) Comparison of growth of *hmbAΔ* and the reconstituted strains to the growth of $hmbA^{+}$ control strain on glucose-sodium nitrate (GLU-NIT) minimal medium. **(b)** Study of the *ER* to various carbon-sources. In all minimal medium, the nitrogen-source was sodium nitrate. The control condition was the GLU (glucose). SUC: sucrose; LAC: lactose; GAL: galactose and ETH: ethanol; GLY: glycerol, MAL: maltose; RAF: raffinose; SOR: sorbitol and XYL: xylose. **(c)** Study of the ER to various nitrogen-sources. In all minimal medium, the carbon-source was glucose. The control condition was the NIT (sodium nitrate). AMM: diammonium L-(+)-tartrate; ALL: allantoin; URE: urea; URI: uric acid; ACE: acetamide and HYP: hypoxanthine. **(d)** Same as panel *c* except that AMM (diammonium L-(+)-tartrate) was used as control condition. **(e)** Study of the *ER* to various temperatures without sorbitol supplementation (UNT, untreated), with osmotic stabilizer (1 M sorbitol) and osmotic stressor (2 M sorbitol). All minimal medium was GLU-NIT, whereas the conditions were as follows: 37 °C UNT in the first and second box plot (37 °C untreated condition), 42 °C UNT in the third box plot (42 °C untreated condition) and 30 °C UNT in the fourth box plot (30 °C

untreated condition). **(f)** Study of the *ER* to the supplementation of the medium with various stressors without (first row of box plots) and with (second row of box plots) an osmotic stabilizer (1 M SOR (sorbitol)). All minimal medium was GLU-NIT. The control condition was the untreated GLU-NIT medium for the first row of box plot, while the control condition for the second row of box plots was GLU-NIT medium supplemented with 1 M sorbitol (1 M SOR). MEN: menadione; CFW: Calcofluor White; SDS: sodium dodecyl sulphate; CRE: congo red; CAF: caffeine and CDS: cadmium sulphate. Concentrations of the stressors are indicated above the box plots. Concentrations of used sole carbon- and nitrogen-sources are listed in the Methods section of the main text. Used strains were *hmbA*$^{+}$ as control (HZS.120); *hmbAΔ* (*hmbA* deletion strain, HZS.320); *C'hmbA* (*hmbAΔ* complemented with *hmbA*, HZS.621), C'*hmbA-gfp* (*hmbAΔ* complemented with *hmbA-gfp*, HZS.371) and C'*NHP6A* (*hmbAΔ* complemented with *NHP6A*, HZS.834). Complete genotypes are listed in the Supplementary Table S2.

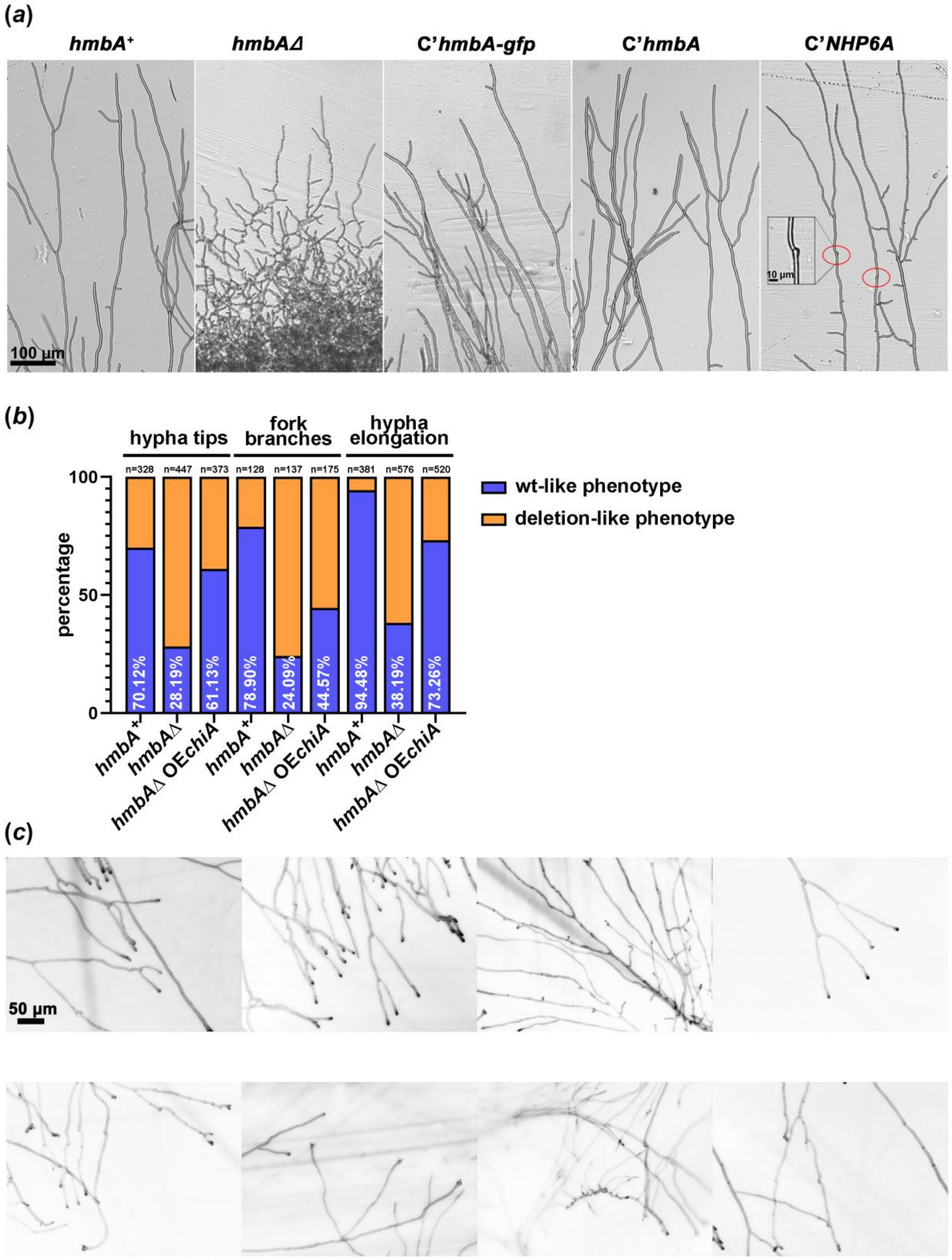

Supplementary Figure S2. Presentation of micromorphology in *hmbA*$^+$ control, *hmbAΔ* and in the various complemented and *chiA* overexpressing strains.

(a) Brightfield images were documented with Leica DMI 4000B, DFC295 detector. Scale bar is shown. Strains were grown on glucose-nitrate minimal medium at 37 °C for 30 h. Red circles denote the relicts of arrested hyphal tips in C'*NHP6A* strain that underwent a germination-like process to continue the polar growth (this phenotype is typical for *hmbAΔ*

strain). Strains were *hmbA*$^+$ as control (HZS.120); *hmbAΔ* (*hmbA* deletion strain, HZS.320); *C'hmbA* (*hmbAΔ* complemented with *hmbA*, HZS.621), C'*hmbA-gfp* (*hmbAΔ* complemented with *hmbA-gfp*, HZS.371); C'*NHP6A* (*hmbAΔ* complemented with *NHP6A*, HZS.834). Complete genotypes are listed in Supplementary Table S2. **(b)** Frequency of occurrence of the wild-type like and deletion-like hypha tips, fork-branches and hypha elongation in *hmbA*$^+$ control (HZS.120), *hmbAΔ* (HZS.320) and in the *chiA* overexpressing *hmbAΔ* strain (*hmbAΔ* OE*chiA*, HZS.921). Complete genotypes are listed in Supplementary Table S2. **(c)** The images show Calcofluor White staining of chitin at the hyphal tips and at the relicts of elongation-arrested hyphal tips in the *hmbAΔ* mutant (HZS.320). Scale bar is shown. Microscopy was carried out with Zeiss Axioobserver 7 Axiocam 503 mono detector with DAPI filter setting.

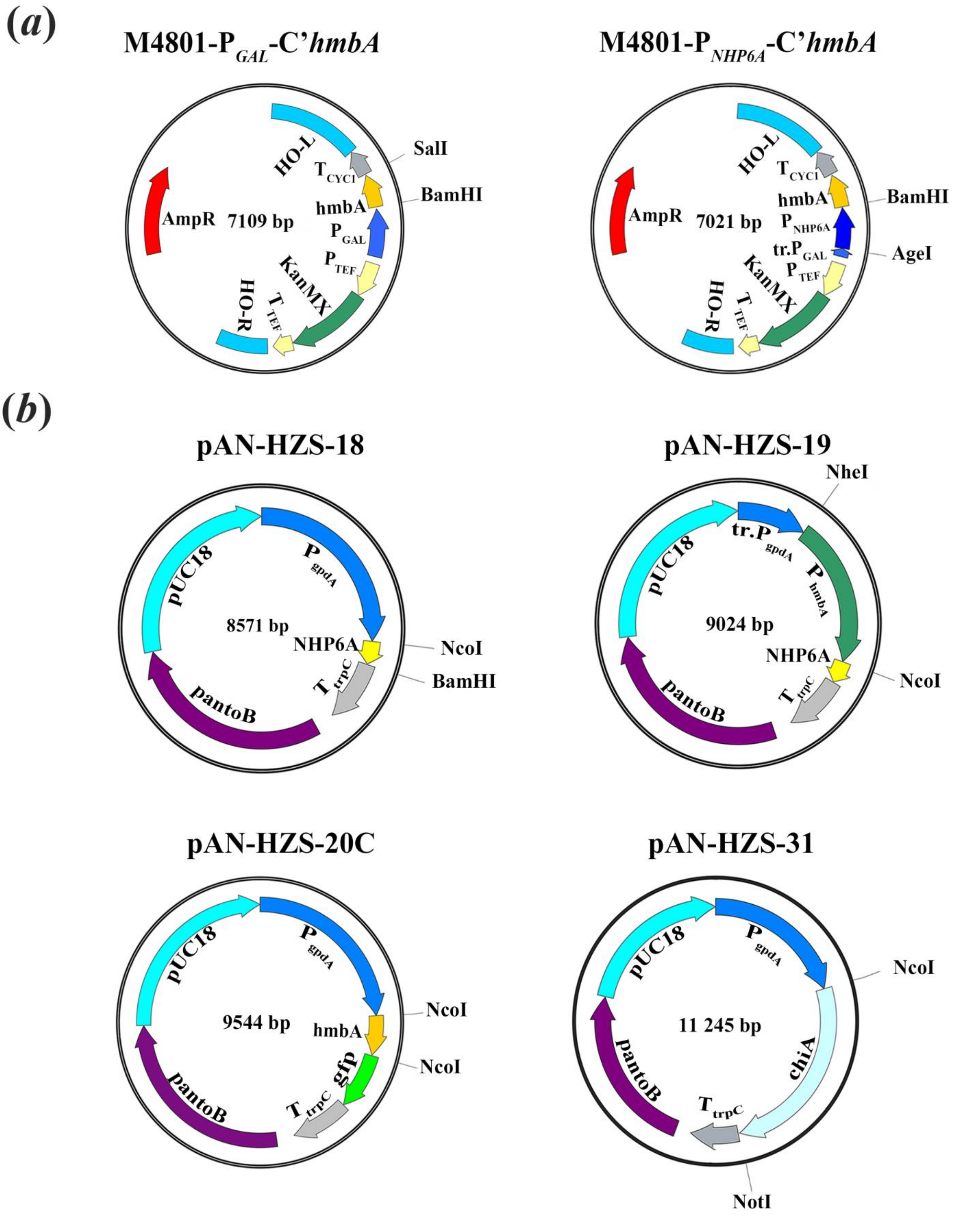

Supplementary Figure S3. Schematic presentation of the vectors constructed in this work.

(*a*) Schematic representation of the yeast transformation vectors developed from the modification of M4801 vector (http://www.addgene.org/51664/). For the detailed description

of the construction process, see Supplementary Methods. Names of the vectors are shown above the circular schemes, the size of the vectors are shown within the circular schemes. Restriction sites used for cloning are shown. Coloured arrows show relevant components of the vectors, the arrowheads indicate the orientation. HO-L and HO-R: left (L) and right (R) arm of the *HO* gene that encodes a DNA endonuclease responsible for mating-type switch via the formation of a double-strand break at the mating-type locus*;* T_{CYC1}: termination sequence of *CYC1* (Cytochrome C1); hmbA: coding gene of HmbA from *A. nidulans*; P_{GAL}: galactose-inducible promoter; P_{TEF} and T_{TEF}: promoter and termination sequence of *TEF* gene (Translational elongation factor EF-1) from *S. cerevisiae;* KanMX: kanamycin resistance marker gene; AmpR: ampicillin resistance marker gene; tr.P_{GAL}:truncated *GAL* promoter; P_{NHP6A}: native *NHP6A* promoter from *S. cerevisiae*. M4801-P_{NHP6A}-C'*hmbA* was used for transformation of HZS.891 and the obtained yeast reconstitution strain was named as yC'*hmbA* (HZS.890). (*b*) Schematic representation of the *A. nidulans* transformation vectors developed from pAN-HZS-1 [2]. For the detailed description of the construction process, see Supplementary Methods. pAN-HZS-18 vector was used for the construction of the transformation vector pAN-HZS-19. pAN-HZS-20C was used as template for the construction of the transformation cassette *hmbA-gfp* generated by using double-joint PCR method (see Supplementary Methods). Unique restriction motifs used for cloning are shown. Coloured arrows show relevant components of the vectors, the arrowheads indicate the orientation. pUC18: standard *E. coli* vector; P_{gpdA}: constitutive promoter of *gpdA* (glyceraldehyde-3-phosphate dehydrogenase coding gene) from *A. nidulans*; gfp: coding gene of Gfp (green fluorescence protein); T_{trpC}: termination sequence of *trpC* gene (tryptophan biosynthesis gene) from *A. nidulans*; pantoB: wild-type *pantoB* gene (coding for pantothenic acid biosynthesis gene) from *A. nidulans*, which serves as selection marker gene for transformation; tr.P_{gpdA}: truncated *gpdA* promoter; hmbA*:* coding gene of HmbA from *A. nidulans;* P_{hmbA}: physiological promoter of *hmbA*;. NHP6A: coding gene of Nhp6Ap from *S. cerevisiae*; chiA: coding gene of ChiA from *A. nidulans.* Names of the vectors are shown above the circular schemes, the size of the vectors are shown within the circular schemes. These vectors were used for transformations of *A. nidulans* recipient strains (see Supplementary Methods) to obtain the C'*NHP6A* (HZS.834) and C'*hmbA-gfp* (HZS.371) *A. nidulans* reconstitution strains and the *chiA* overexpression *hmbA*Δ OE*chiA* strain (HZS.834).

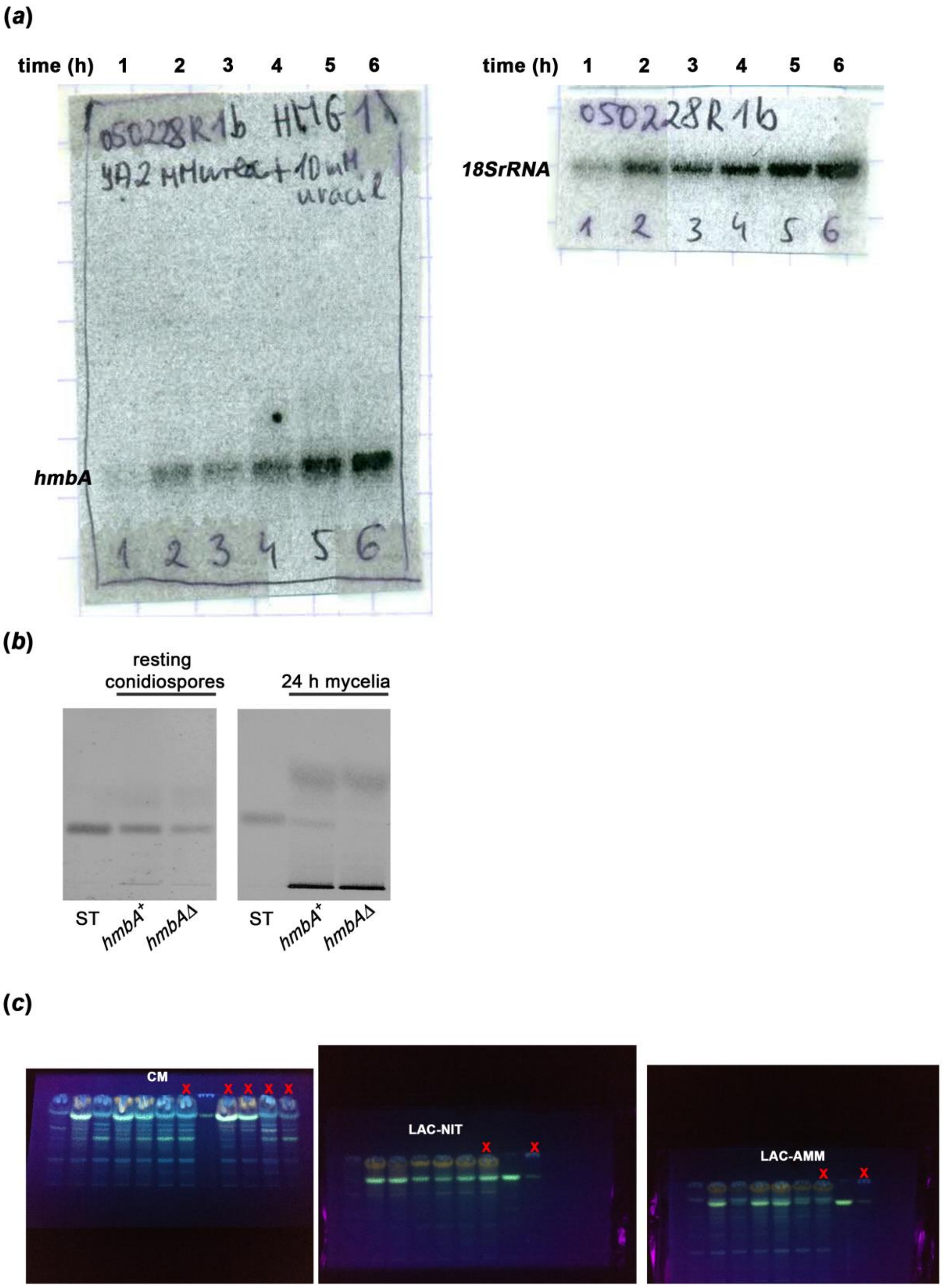

Supplementary Figure S4: Original images of Northern blots and TLC plates presented in Figures 4a, 7b and 8.

(a) Original images of Northern blot results presented in Figure 4a. [^{32}P]-dCTP labelled *hmbA* (left) and *18S rRNA* gene-specific DNA molecules (right) were used as gene probes and the [^{32}P]-dCTP signals (1011 nt and 1674 nt for hmbA and 18S rRNA RNAs, respectively) were documented by PhosphorImager (Molecular Dynamics, Sunnyvale, CA) using IMAGE

QUANT software, version 3.3 (Molecular Dynamics). The visualized images were printed and archived. The original images presented here in this figure are scanned copies of the printed archived images. In the case of the 18S rRNA blot, the filter was cut prior hybridization.

b) Original images of TLC plates presented in Figure 7b.

c) Original images of TLC plates presented in Figure 8. Red X denote lanes, which were cut from the original TLC images. The other lanes with the detailed description confer to those presented and described in Figure 8.

SUPPLEMENTARY TABLES

for

The role of the *Aspergillus nidulans* High Mobility Group B protein HmbA, the orthologue of *Saccharomyces cerevisiae* Nhp6p

by

Judit Ámon[1+], Gabriella Varga[1+], Ilona Pfeiffer[1], Zoltán Farkas[2], Zoltán Karácsony[1#], Zsófia Hegedűs[1], Csaba Vágvölgyi[1], and Zsuzsanna Hamari[1*]

[1]University of Szeged Faculty of Science and Informatics, Department of Microbiology, Szeged, Hungary

[2]Synthetic and Systems Biology Unit, Institute of Biochemistry, Biological Research Centre, Eötvös Loránd Research Network, Szeged, Hungary

*Corresponding author:
Zsuzsanna Hamari
hamari@bio.u-szeged.hu

Content:

Supplementary Table S1: Results of HmbA and Nhp6Ap protein modelling and superimposition of these models

Supplementary Table S2: List of *A. nidulans* and *S. cerevisiae* strains used in this work.

Supplementary Table S3. List of compounds and concentrations used in the various media

Supplementary Table S4: List of primers used in this study.

Supplementary Table S1. Results of HmbA and Nhp6Ap protein modelling and superimposition of these models

**Quality assessment of the protein models**	**HmbA (106 AAs)**	**Nhp6Ap (93 AAs)**
C-score of initial model	1.31	0.28
estimated TM-score of initial model	0.55±0.15	0.68±0.12
estimated RMSD of initial model	6.8±4.0 Å	4.4±2.9 Å
TM-score of refined model to initiate model	0.9881	0.9935
RMSD of refined model to initiate model	0.446 Å	0.285 Å
RAMA[1], % of AAs in favoured region	93.3% (84 AAs)	96.3% (78 AAs)
RAMA[1], % of AAs in allowed region	4.4% (4 AAs)	1.2% (1 AA)
RAMA[1], % of AAs in disallowed region	2.2% (2 AAs)	2.5% (2 AAs)
number of non-Pro, non-Gly residues	90 AAs	81 AAs
**Quality assessment of the superimposition of the modelled HmbA and Nhp6Ap proteins (by built-in Matchmaker of UCSF Chimera 1.14)**		
Sequence alignment scores[2]	361.5	
Structure RMSDs between 75 pruned atom pairs in superimposition	0.509 Å	
Structure RMSDs between all 93 atom pairs in superimposition	4.725 Å	
Structure RMSDs across all 89 fully populated columns in the final alignment	1.248 Å (SDM (cutoff 5.0): 24.766; Q-score: 0.685)	
modelled interacting molecule in the PDB model	DNA	
Overall identity between two proteins	67.74%	

[1] RAMA: Ramachandran plot analysis results on non-Proline and non-Glycine regions derived from using Procheck server (https://servicesn.mbi.ucla.edu/PROCHECK/)

[2] Computing secondary structure assignments of superimposed models used ksdssp (Kabsch and Sander Define Secondary Structure of Proteins) with the following parameter values: -0.5 energy cutoff; minimum helix length 3; minimum strand length 3. Sequence alignment scores were obtained by Matchmaker (built in UCSF Chimera 1.14) with the following parameter values: chain pairing: bb; alignment algorithm: Needleman-Wunsch using BLOSUM-62 matrix; ss (secondary structure) fraction: 0.3; gap opening penalties (HH/SS/other)(HH: intra Helix; SS: intra Strand) 18/18/6, gap extension penalty: 1; ss scoring matrix: (O, S): -6 (H, O): -6 (H, H): 6 (S, S): 6 (H, S): -9 (O, O): 4 (H is Helix, S is Strand, O is Other); iteration cutoff: 2.

Supplementary Table S2. List of *A. nidulans* and *S. cerevisiae* strains used in this work.

All *Aspergillus* strains listed are *veA1* mutant.

***A. nidulans* strains**			
Strain	Genotype	Purpose	Reference
HZS.117	*yA2*	Northern blot	this work
HZS.120	*riboB2 pabaA1*	growth test; microscopic experiments; mRNA expression analysis; metabolite analysis	[3]
HZS.145	*veA1*	template DNA for PCRs	[4]
HZS.320	*hmbAΔ::riboB*$^{+}$ *pantoB100 pabaA1 riboB2*	recipient strain for transformation experiment to obtain C'*NHP6A*, C'*hmbA-gfp* and *hmbAΔ* OE*chiA* strains; growth test; microscopic experiments; mRNA expression analysis; metabolite analysis	[5]
HZS.371	*hmbAΔ::riboB*$^{+}$ *pantoB100 pabaA1 riboB2* +*in trans* "*hmbA-gfp* cassette" in 2 copy	microscopic experiments; growth test; metabolite analysis	this work (by transformation of the "*hmbA -gfp* cassette" into HZS.320)
HZS.621	*hmbAΔ::riboB*$^{+}$ *pantoB100 pabaA1 riboB2* +pAN-HZS-9 plasmid integrated into the *hmbA* promoter in 1 copy	growth test; metabolite analysis	[5]
HZS.834	*hmbAΔ::riboB*$^{+}$ *pantoB100 pabaA1 riboB2* + *in trans* pAN-HZS-19 plasmid in 1 copy	growth test; metabolite analysis	this work (by transformation of the pAN-HZS-19 plasmid into HZS.320)
HZS.921	*hmbAΔ::riboB*$^{+}$ *pantoB100 pabaA1 riboB2* +*in trans* pAN-HZS-31	microscopic experiments; metabolite analysis	this work (by transformation of the pAN-HZS-31 plasmid into HZS.320)
***S. cerevisiae* strains**			
Strain	Genotype	Purpose	Reference
NHP6AΔ	*nhp6aΔ::KanMX his3Δ1*	recipient strain for	[6]

	leu2Δ0 met15Δ0 ura3Δ0 MAT-a	transformation experiment to obtain NHP6AΔ (HphMX) strain	
NHP6BΔ	*nhp6bΔ::NatMX can1Δ::p$_{STE2}$-SpHis5 lyp1Δ his3Δ1 leu2Δ0 ura3Δ0 met15Δ0 MAT- α*	parental strain in genetic crosses with NHP6AΔ (HphMX)	[7]
NHP6AΔ (HphMX)	*nhp6aΔ::HphMX his3Δ1 leu2Δ0 met15Δ0 ura3Δ0 MAT-a*	parental strain in genetic crosses with NHP6BΔ	this work (by transformation of the pCRII-TOPO::hphMX plasmid into NHP6AΔ strain)
HZS.890	*nhp6aΔ::HphMX nhp6bΔ::NatMX his3Δ1 leu2Δ0 met15Δ0 ura3Δ0 +in trans* M4801-P$_{NHP6A}$-C'*hmbA* plasmid in 1 copy	mRNA expression analysis; growth test; fitness measurements	this work (by transformation of the M4801-P$_{NHP6A}$-C'*hmbA* plasmid into HZS.891)
HZS.891	*nhp6aΔ::HphMX nhp6bΔ::NatMX his3Δ1 leu2Δ0 met15Δ0 ura3Δ0*	recipient strain for transformation experiment to obtain yC'*hmbA* strain; mRNA expression analysis; growth test; fitness measurements	this work (obtained by genetic cross of NHP6AΔ (HphMX) and NHP6BΔ)
Y199	*his3Δ::KanMX hoΔ::NatMX his3Δ1 leu2Δ0 met15Δ0 ura3Δ0 MAT a*	mRNA expression analysis; growth test; fitness measurements	provided by Z. Farkas

Explanation of mutant alleles, which are not described in the text: *veA1* is a mutation in the *veA* gene resulting profuse conidiation regardless of the presence or absence of light [8]. T_{trpc}: terminator sequence of trpC gene. *KanMX* (G418), *NatMX* (nourseothricin) and *HphMX* (hygromycin B) symbols are resistance marker genes. *lyp1Δ:* deletion of a lysine permease responsible for uptake of cationic amino acids. *hoΔ:* deletion of HO (YDL227C) made by allelic replacement using the *ho::KanMX* deletion cassette. *HO* is a gene encoding a DNA endonuclease responsible for mating-type switch *via* the formation of a double-strand break at the mating-type locus. *can1Δ*: deletion of *CAN1* (YEL063C) made by allelic replacement using the *can1::p$_{STE2}$-SpHis5* deletion cassette. *CAN1* is a gene encoding arginine permease and its deletion confers resistance to the toxic arginine analog, canavanine. The selectable marker, in this case, is the p$_{STE2}$-*SpHis5*, which is the His5p of *Schizosaccharomyces pombe* under the control of the promoter of *STE2* (p$_{STE2}$), that is active only in MAT-a haploid *S. cerevisiae* cells. Other gene symbols refer to auxotrophies: *pabaA1*, p-aminobenzoic acid; *pantoB100*, pantothenic acid and *riboB2*, riboflavin; *his3Δ1*: histidine; *leu2Δ0*: leucine; *met15Δ0*: methionine; *ura3Δ0*: uracil, uridin.

Supplementary Table S3. List of compounds and concentrations used in the various media

Compounds	Used concentration
**Carbon sources**	
glucose (GLU), sucrose (SUC), galactose (GAL), xylose (XYL), maltose (MAL), lactose (LAC) and sorbitol (SOR)	1% (m/V)
ethanol (ETH) and glycerol (GLY)	2% (V/V)
raffinose (RAF)	3.5% (m/V)
**Nitrogen sources**	
sodium-nitrate (NIT) and acetamide (ACE)	10 mM
diammonium L-(+)-tartrate (AMM) and urea (URE)	5 mM
allantoin (ALL) and hypoxanthine (HYP)	1 mM
uric acid (URI)	0.6 mM
**Stressors**	
caffeine (CAF)	1 mM
Calcofluor White (CFW)	10 µM
sodium dodecyl sulphate (SDS)	35 µM
congo red (CRE), menadione (MEN), cadmium-sulphate (CDS)	50 µM

Supplementary Table S4. List of primers used in this study.

**C'*NHP6A* strain**	
nhp6A NcoI frw	5'-tttttttccatggtcaccccaagagaacctaag-3'
nhp6A BamHI rev	5'-tttttttggatccctaagccaaagtggcgttatataactc-3'
hmbA prom NheI frw	5'-tttttttgctagcgatcctcaatgaaccttgcccttg-3'
hmbA prom NcoI rev	5'-tttttttccatggggtgaaggtctgaagctgttggacg-3'
**C'*hmbA-gfp* strain**	
hmbA NcoI frw	5'-tttttttccatggctaaggccaatcctac-3'
hmbA linker NcoI rev	5'-tttttttccatggaatcaagatcgactgtatcaataagggacgactcctcatcctcttcg-3'
hmbA upst chim frw2	5'-*cttcgtccaacagcttcagaccttcacca*tggctaaggccaatcctacccg-3'
pantoB hmbA down chim rev	5'-*cggaggtcaagcgactcgacactaaggtg*acataatcttatgatccataccacctagc-3'
hmbA upst frw	5'-ctctgacctgccacgaggccttgctctatg-3'
hmbA upst rev	5'-ggtgaaggtctgaagctgttggacgaagag-3'
hmbA down frw	5'-caccttagtgtcgagtcgcttg-3'
hmbA down rev	5'-gcatacaatgcgagcacggtggtcgtcatc-3'
hmbA upst nest frw	5'-ggagacatttcgaactgtatcagggctaac-3'
hmbA down nest rev	5'-cggtgctgttgctgcttgaggacgaggag-3'
hmbAΔ* OE*chiA	
chiA NcoI fw	5'-tttttttccatggcccctaaactgtttaccttc-3'
chiA NotI rev	5'-tttttttgcggccgcttacaaaacagcaagcagggagag-3'
**yC'*hmbA***	
c-hmbA BamHI frw	5'-tttttttggatccatgcctaaggccaatcctacc-3'
c-hmbA SalI rev	5'-tttttttgtcgacttaggacgactcctcatcctcttc-3'
NHP6A prom AgeI frw	5'-tttttttaccggttcttggagcgttgagcacgtctac-3'
NHP6A prom BamHI rev	5'-tttttttggatcctgcgactgtgctttactatgtatagggtag-3'
**Checking gene deletions and integrations**	
NHP6A confA frw	5'-cacgacgttaaatactgttcaagtg-3'
NHP6B confB frw	5'-ccacctctcacccaatattctg-3'
KanB rev	5'-ctgcagcgaggagccgtaat-3'
NHP6A prom AgeI frw	5'-tttttttaccggttcttggagcgttgagcacgtctac-3'
NHP6A prom BamHI rev	5'-tttttttggatcctgcgactgtgctttactatgtatagggtag-3'
**qPCR**	
hmbA ReTi frw	5'-aaagatgctcggtgagaagtg-3'
hmbA ReTi rev	5'-ctcgtaccgcttcttgtcag-3'
NHP6A ReTi frw	5'-agaagttgggtgagaagtgg-3'
NHP6A ReTi rev	5'-ttcatatctcttcttatcggcctg-3'
gfp ReTi frw	5'-atcttcttcaaggacgacgg-3'
gfp ReTi rev	5'-ttgaagtcgatgcccttcag-3'
chiA ReTi frw	5'-ctcttcaagcacttccactc-3'
chiA ReTi rev	5'-gccagatgatgtacttgtagag-3'
pantoB ReTi frw	5'-gttaagagccgagcgtatcc-3'
pantoB ReTi rev	5'-cttcaggtaattcatcaacagcc-3'
actA ReTi frw2	5'-accatgtaccctggtatctc-3'

actA ReTi rev2	5'-ggaggagcaatgatcttgac-3'
UBC6 ReTi frw	5'-ttacaagggcggtcaatatcac-3'
UBC6 ReTi rev	5'-gggtggtaatcactcatagaaagg-3'
**RT-qPCR**	
actA ReTi frw	5'-ggtatcatgatcggtatggg-3'
actA ReTi rev	5'-tatctgagtgtgaggatacca-3'
UBC6 ReTi frw	5'-ttacaagggcggtcaatatcac-3'
UBC6 ReTi rev	5'-gggtggtaatcactcatagaaagg-3'
chiA ReTi rev	5'-gccagatgatgtacttgtagag-3'
chiA ReTi frw	5'-ctcttcaagcacttccactc-3'
SNR6 ReTi frw	5'-cgaagtaacccttcgtggac-3'
SNR6 ReTi rev	5'-aacggttcatccttatgcagg-3'

Underlined letters in the primer sequences refer to the restriction sites designed within.
Italic letters at the 5' end refer to the chimeric nature of the primer.

SUPPLEMENTARY METHODS

for

The role of the *Aspergillus nidulans* High Mobility Group B protein HmbA, the orthologue of *Saccharomyces cerevisiae* Nhp6p

by

Judit Ámon[1+], Gabriella Varga[1+], Ilona Pfeiffer[1], Zoltán Farkas[2], Zoltán Karácsony[1#], Zsófia Hegedűs[1], Csaba Vágvölgyi[1], and Zsuzsanna Hamari[1*]

[1]University of Szeged Faculty of Science and Informatics, Department of Microbiology, Szeged, Hungary

[2]Synthetic and Systems Biology Unit, Institute of Biochemistry, Biological Research Centre, Eötvös Loránd Research Network, Szeged, Hungary

*Corresponding author:

Zsuzsanna Hamari

hamari@bio.u-szeged.hu

Content:

Construction of *S. cerevisiae nhp6AΔBΔ* double deletion mutant

Construction of *hmbA* expressing *nhp6AΔBΔ* yeast strain

Construction of *NHP6A*, *hmbA-gfp* and *chiA* expressing *hmbAΔ* strains

Extraction and detection of sterigmatocystin (STC) by TLC and HPLC

Extraction and detection of trehalose and glycerol by TLC and HPLC

Construction of *S. cerevisiae nhp6AΔBΔ* double deletion mutant

To generate a double deletion strain for *NHP6A* and *NHP6B*, first we obtained the *nhp6aΔ* (*nhp6aΔ::KanMX, his3Δ1 leu2Δ0 met15Δ0 ura3Δ0, MAT-a*) strain from the YKO Mat-a collection [6] and the *nhp6bΔ* strain (*nhp6bΔ::NatMX, can1Δ::*P_{STE2}*-SpHis5 lyp1Δ his3Δ1 leu2Δ0 ura3Δ0 met15Δ0, MAT-*α) from the SGA (Synthetic Genetic Array) query collection [7]. To be able to use an expression construct at a later stage, we first swapped the KanMX deletion cassette of the *nhp6aΔ* strain to HphMX marker as follows. We performed a HindIII-EcoRI digestion of the pCRII-TOPO::hphMX plasmid and then after heat-inactivation, we transformed the plasmid into the *nhp6aΔ* strain using the standard lithium acetate method [9]. Transformants were selected on YPD supplemented with hygromycin (PAA Laboratories) at a final concentration of 400 μg/ml. The loss of the KanMX marker was confirmed by testing the inability of the transformants to grow on YPD supplemented with G418 (Sigma) at a final concentration of 200 μg/ml. We followed an established protocol [7] to generate the double mutant *nhp6AΔBΔ* by genetic cross. After several steps of selection, we used *hygromycin* and nourseothricin supplemented (400 *μg/ml* and 100 *μg/ml*, respectively) haploid selection medium to isolate MAT-a haploid double deletion mutants (*nhp6aΔ::HphMX, nhp6bΔ::NatMX*). Gene deletion was checked by "NHP6A confA frw"- "KanB rev" and "NHP6B confB frw"- "KanB rev" primers. Primers are listed in Supplementary Table S4.

Construction of *hmbA* expressing *nhp6AΔBΔ* yeast strain

In order to obtain the *hmbA* expressing *nhp6AΔBΔ* strain (yC'*hmbA*), the 321 bp cDNA of *A. nidulans hmbA* gene was ampified by "c-hmbA BamHI frw" and "c-hmbA SalI rev" primers and the BamHI-SalI digested PCR product was cloned into a BamHI-SalI digested yeast expression vector, M4801 (http://www.addgene.org/51664/). The P_{GAL} promoter of the obtained vector (M4801-P_{GAL}-C'*hmbA*) (Supplementary Fig. S3*a*) was truncated by AgeI-BamHI digestion and the 308 bp long promoter sequence of *NHP6A* (P_{NHP6A}) was amplified (using "NHP6A prom AgeI frw" and "NHP6A prom BamHI rev" primers) and cloned into the AgeI-BamHI digested M4801-P_{GAL}-C'*hmbA* vector. The resulted vector (M4801-P_{NHP6A}-C'*hmbA*) (Supplementary Fig. S3*a*) was transformed into *nhp6AΔBΔ* strain (HZS.891) after NotI digestion using the standard lithium acetate method [9]. Correct transformants where the expression plasmid was integrated into the HO locus were selected on YPD supplemented

with hygromycin, nourseothricin and G418 at a final concentration of 400 µg/ml, 200 µg/ml and 200 µg/ml, respectively. Integration of the vector was checked by "NHP6A prom AgeI frw"-"c-hmbA SalI rev" primers, while the copy number of integration events were checked with quantitative PCR according to Herrera et al. [10] using "hmbA ReTi frw"- "hmbA ReTi rev" primers and the single copy control "UBC6 ReTi frw"- "UBC6 ReTi rev" primers. Primers are listed in Supplementary Table S4.

Construction of *NHP6A, hmbA-gfp* and *chiA* expressing *hmbAΔ* strains

Transformation cassettes were constructed by using double-join PCR (DJ-PCR) method [11] and cloning. In order to obtain *NHP6A* complemented *hmbAΔ* strain (C'*NHP6A*), *NHP6A* gene was amplified from *S. cerevisiae* genome by using "nhp6A NcoI frw" and "nhp6A BamHI rev" primer pair (282 bp). The PCR product was digested with NcoI-BamHI and cloned into NcoI-BamHI digested pAN-HZS-1 vector [17] (the digestion eliminated the *gfp* gene from the vector). The obtained vector (pAN-HZS-18) expressed the *NHP6A* from the constitutive promoter P_{gpdA}. In order to express *NHP6A* at the physiological level similar to that of *hmbA*, the P_{gpdA}. promoter was truncated by NheI-NcoI digestion in pAN-HZS-18 that was followed by the cloning of the NheI-NcoI digested PCR product of the 1863 bp long promoter region of *hmbA* (amplified by "hmbA prom NheI frw" and "hmbA prom NcoI rev" primers). The obtained *NHP6A* expressing vector (pAN-HZS-19) (Supplementary Fig. S3*b*) was used for transformation of *hmbAΔ* strain (HZS.320).

In order to obtain *hmbA-gfp* expressing strain (C'*hmbA-gfp*), the *hmbA* gene without stop codon and with an 8 amino acids coding linker sequence at the 3'end (LIDTVDLD) was amplified from wild-type *A. nidulans* genome (HZS.145) by "hmbA NcoI frw" and "hmbA linker NcoI rev" primers and cloned into NcoI site of pAN-HZS-1 vector [2]. The resulted vector (pAN-HZS-20C) (Supplementary Fig. S3*b*) was used as a template to amplify the 4797 bp long *hmbA-gfp* gene phusion followed by the 3' UTR of the *trpC* gene (T_{trpC}) and the *pantoB* selection marker gene by using the chimeric primer pair "hmbA upst chim frw2" and "pantoB hmbA down chim rev". The *hmbA* –linker – *gfp* - T_{trpC} - *pantoB* carrying PCR product was joined to the PCR amplified 3428 bp long upstream ("hmbA upst frw"-"hmbA upst rev") and 3058 bp long downstream ("hmbA down frw"-„hmbA down rev") regions of *hmbA* by using DJ-PCR method with nested primer pair ("hmbA upst nest frw"-"hmbA down

nest rev") and wild-type *A. nidulans* genome (HZS.145) as a template. The assembled PCR product of *hmbA-gfp* cassette was used to transform the *hmbAΔ* strain (HZS.320).

In order to obtain *chiA* overexpressing *hmbAΔ* strain (*hmbAΔ* OE*chiA*), the 2935 bp long *chiA* gene was amplified from wild-type *A. nidulans* genome (HZS.145) by "chiA NcoI fw" and "chiA NotI rev" primer pair. The NcoI-NotI digested PCR product was cloned into NcoI-NotI digested pAN-HZS-1 vector [2] (the digestion eliminated the *gfp* gene from the vector). The resulted vector (pAN-HZS-31) was used to transform the *hmbAΔ* strain (HZS.320) (Supplementary Fig. S3*b*).

After transformation of *hmbAΔ* (HZS.320) with pAN-HZS-9, pAN-HZS-19, the *hmbA-gfp* cassette and pAN-HZS-31, pantothenic acid prototroph transformant strains were collected and the copy number of integration events was measured by qPCR using *hmbA*, *NHP6A*, *gfp*, *chiA* and *pantoB* specific primer pairs ("hmbA ReTi frw"-"hmbA ReTi rev", "NHP6A ReTi frw"-"NHP6A ReTi rev", "gfp ReTi frw"-"gfp ReTi rev", "chiA ReTi frw"-"chiA ReTi rev" and "pantoB ReTi frw"-"pantoB ReTi rev", respectively). The γ-actin coding *actA* (AN6542) was used as reference single copy gene (amplified by "actA ReTi frw2"-"actA ReTi rev2" primers). The copy number was calculated as described by Herrera et al. [10]. The verified transformants C'*hmbA-gfp* (HZS.371, with two copies of transgene), C'*NHP6A* (HZS.834, with one copy of transgene) and *hmbA*Δ OE*chiA* (HZS.921, with one copy of transgene) were further used in the experiments. Complete genotypes of the strains and all the used primers are listed in Supplementary Tables S2 and S4, respectively.

Extraction and detection of sterigmatocystin (STC) by TLC and HPLC

Five agar blocks were excised from the centre of 6-day-old *A. nidulans* colonies with a cork borer with 10 mm diameter. STC was extracted from the agar blocks by using 10 ml chloroform. The extracts were concentrated into 1 ml final volume by heating the samples to 65 °C. Samples were loaded on Kieselgel 60 (Merck) plates after being normalized for the protein content of the samples (by using Bradford reagent) and the chromatogram was developed in toluol : ethylacetate : formic acid (50:40:10 V/V/V). Secondary metabolites were detected and recorded under UV light (366 nm), after being sprayed with 10% $AlCl_3$ in ethanol, and heated to 100 °C for 1 min. Identification of STC was accomplished by using an STC standard (Sigma).

HPLC detection of STC was carried out using a Shimadzu HPLC system (Shimadzu, Kyoto, Japan) equipped with an SPD-10Avp UV-VIS detector, an LC-20AD binary pump, a SIL-20A autosampler, a DGU-14A degasser, a CTO-10ASvp column thermostat, and a CBM-20A system controller. For data acquisition and evaluation Class VP ver. 6.2 software was used. The separation of sterigmatocystin was performed on a Purosphere Star RP18e, 250 × 4mm, 5 μm column (Merck KGaA, Darmstadt, Germany) at a column temperature of 40 °C. The injected sample volume was 5 μl with a flow rate of 0.5 ml/min. Mixture of water (component A) and methanol (component B) were used as eluents with a gradient program starting with an isocratic step at 60% component B for 1 minute, then increasing to 80% component B in 8 minutes followed by another isocratic step for 16 minutes at 80% component B. After reaching the initial solvent composition in 1 minute, it was held for re-equilibration for 11 minutes. The peak of sterigmatocystin was detected at $\lambda = 254$ nm.

Extraction and detection of trehalose and glycerol by TLC and HPLC

For the monitoring of metabolism of conidial trehalose to glycerol during the swelling of conidiospores that precedes the germ tube formation, 10^9 conidiospores were inoculated into 200 ml of minimal medium and cultivated at 37 °C with 180 rpm shaking. 40 ml samples were taken after 0, 30, 60, 90, 120 min and 24 h of incubation. The conidia were collected by centrifugation (13,000 g, 10 min) and dissolved in 1 ml of 5% (m/V) trichloroacetic acid (TCA). Spores were counted by the use of hemocytometer, whereas the dry weight of the mycelia was measured for the purpose of the normalization of measured trehalose/glycerol content. Sugars and polyalcohols were extracted two times with 1 ml of 5% (m/V) TCA at 80 °C. The solid fractions were removed by centrifugation (13,000 g, 10 min) and the liquid fractions were pooled and concentrated under vacuum to the final volume of 500 μl.

Samples were loaded on Kieselgel 60 (Merck) plates after being normalized for the conidiospore number or mycelial dry weight and the chromatogram was developed in chloroform : methanol : distilled water : acetic acid (55:33:8:1 V/V/V/V). Trehalose was detected by submerging the plates in phenol/sulphuric acid solution (3 g phenol, 5 ml sulphuric acid, 95 ml ethanol) followed by incubation at 100 °C for 10 min. Trehalose standard (Fluka) was used as control.

In case of the HPLC analysis the samples were analysed in separated chromatographic runs for both glycerol and trehalose contents by using the Shimadzu HPLC system as described

above (Shimadzu, Kyoto, Japan) except that the original detector was replaced with a refractive index detector (RID-10). In the case of glycerol, the stationary phase was a Hi-Plex H column (300 x 7.7 mm; Agilent, USA) and the mobile phase was 0.005 M H_2SO_4 solution at a flow rate of 0.4 ml/min. During the trehalose analysis, a silica Si 100 column was used (250 × 4.6 mm; Serva Feinbiochemica, Germany) and the components were eluted with 17/83 V/V mixture of water and acetonitrile at the flow rate of 1.2 ml/min. In both cases, the injection volume was 20 µl and the cell of the detector was tempered at 55 °C. The run time of the glycerol and trehalose HPLC methods was 30 min and 15 min, while the column temperature was maintained at 60 °C and 35 °C, respectively. The retention time of glycerol and trehalose peaks in the two separated method were 19.9 min and 7.3 min, respectively.

SUPPLEMENTARY REFERENCES

1 R Core Team. *R: A language and environment for statistical computing.*, <https://www.R-project.org/> (2021).

2 Karacsony, Z., Gacser, A., Vagvolgyi, C., Scazzocchio, C. & Hamari, Z. A dually located multi-HMG-box protein of *Aspergillus nidulans* has a crucial role in conidial and ascospore germination. *Molecular microbiology* **94**, 383-402, doi:10.1111/mmi.12772 (2014).

3 Hamari, Z. *et al.* Convergent evolution and orphan genes in the Fur4p-like family and characterization of a general nucleoside transporter in *Aspergillus nidulans*. *Molecular microbiology* **73**, 43-57, doi:10.1111/j.1365-2958.2009.06738.x (2009).

4 Amon, J. *et al.* A eukaryotic nicotinate-inducible gene cluster: convergent evolution in fungi and bacteria. *Open Biol* **7**, 170199, doi:10.1098/rsob.170199 (2017).

5 Bokor, E. *et al.* HMGB proteins are required for sexual development in *Aspergillus nidulans*. *PLoS One* **14**, e0216094, doi:10.1371/journal.pone.0216094 (2019).

6 Giaever, G. *et al.* Functional profiling of the *Saccharomyces cerevisiae* genome. *Nature* **418**, 387-391, doi:10.1038/nature00935 (2002).

7 Tong, A. H. & Boone, C. Synthetic genetic array analysis in *Saccharomyces cerevisiae*. *Methods Mol Biol* **313**, 171-192, doi:10.1385/1-59259-958-3:171 (2006).

8 Kafer, E. Origins of translocations in *Aspergillus nidulans*. *Genetics* **52**, 217-232 (1965).

9 Gietz, R. D. & Schiestl, R. H. High-efficiency yeast transformation using the LiAc/SS carrier DNA/PEG method. *Nat Protoc* **2**, 31-34, doi:10.1038/nprot.2007.13 (2007).

10 Herrera, M. L., Vallor, A. C., Gelfond, J. A., Patterson, T. F. & Wickes, B. L. Strain-dependent variation in 18S ribosomal DNA Copy numbers in *Aspergillus fumigatus*. *J Clin Microbiol* **47**, 1325-1332, doi:10.1128/JCM.02073-08 (2009).

11 Yu, J. H. *et al.* Double-joint PCR: a PCR-based molecular tool for gene manipulations in filamentous fungi. *Fungal genetics and biology : FG & B* **41**, 973-981, doi:10.1016/j.fgb.2004.08.001 (2004).
